# Early stratification of radiotherapy response by activatable inflammation magnetic resonance imaging

**DOI:** 10.1038/s41467-020-16771-y

**Published:** 2020-06-15

**Authors:** Zijian Zhou, Hongzhang Deng, Weijing Yang, Zhantong Wang, Lisen Lin, Jeeva Munasinghe, Orit Jacobson, Yijing Liu, Longguang Tang, Qianqian Ni, Fei Kang, Yuan Liu, Gang Niu, Ruiliang Bai, Chunqi Qian, Jibin Song, Xiaoyuan Chen

**Affiliations:** 1 Laboratory of Molecular Imaging and Nanomedicine, National Institute of Biomedical Imaging and Bioengineering, National Institutes of Health, Bethesda, MD 20892 USA; 2 MOE Key Laboratory for Analytical Science of Food Safety and Biology, College of Chemistry, Fuzhou University, Fuzhou, 350116 Fujian China; 3 Laboratory of Functional and Molecular Imaging, National Institute of Neurological Disorders and Stroke, National Institutes of Health, Bethesda, MD 20892 USA; 4 Interdisciplinary Institute of Neuroscience and Technology, School of Medicine, Zhejiang University, Hangzhou, 310029 China; 5 Department of Radiology, Michigan State University, East Lansing, MI 48824 USA

**Keywords:** Magnetic resonance imaging, Cancer imaging, Nanoparticles

## Abstract

Tumor heterogeneity is one major reason for unpredictable therapeutic outcomes, while stratifying therapeutic responses at an early time may greatly benefit the better control of cancer. Here, we developed a hybrid nanovesicle to stratify radiotherapy response by activatable inflammation magnetic resonance imaging (aiMRI) approach. The high Pearson’s correlation coefficient *R* values are obtained from the correlations between the *T*_1_ relaxation time changes at 24–48 h and the ensuing adaptive immunity (*R* = 0.9831) at day 5 and the tumor inhibition ratios (*R* = 0.9308) at day 18 after different treatments, respectively. These results underscore the role of acute inflammatory oxidative response in bridging the innate and adaptive immunity in tumor radiotherapy. Furthermore, the aiMRI approach provides a non-invasive imaging strategy for early prediction of the therapeutic outcomes in cancer radiotherapy, which may contribute to the future of precision medicine in terms of prognostic stratification and therapeutic planning.

## Abstract (web summary)

Non-invasive approaches to stratify early responses to therapy are of great value to improve cancer patient management. Here the authors design a hybrid nanovesicle-based activatable inflammation magnetic resonance method for the early prediction of response to radiotherapy.

## Introduction

Early prediction of cancer treatment efficacy is of great value to cancer patients^1,2^. Although there are many strategies and combination systems available for the deployment of cancer therapy in the clinic, successful cancer control is still underrepresented^3^. Mounting evidence indicates that cancer patients have varying degree of responses to different treatments due to tumor heterogeneity, which makes the treatment planning imperative considering the fast progressive nature of cancers^4,5^. One would expect that therapeutic efficacy can be predicted at an early time of cancer treatment, which will greatly benefit cancer patients in terms of reducing side toxicity from ineffective treatments, and more importantly, earning time for better treatment optimization^6^. However, it is technically demanding and challenging to determine whether a given treatment will be effective or not at an early time, which could therefore lead to a devastating outcome if the tumor growth is not controlled before its invasion and metastasis. The biopsy-based biomarker detection methods have been utilized to monitor therapeutic response but are subject to errors and limitations of invasive tissue collection^2^. Therefore, non-invasive and accurate methods enabling to gain insights of therapeutic response are highly desirable for better management of cancer therapy^7–9^.

Radiation therapy (RT), referring to external-beam X-ray irradiation hereafter, has gained acceptance for treating over 50% cancer patients in clinic. RT can lead to DNA damage in tumor cells that mediate the dysregulation of tissue resolution and homeostasis, resulting in the development of late adverse effects (e.g., vasculopathy and hypoxia)^10,11^. Unfortunately, the effective RT is largely compromised due to heterogeneous radiation responses and radioresistance, which vary in different cancer types and among individuals^12^. Therefore, early assessment of RT response from biological side has received much attention through evaluating vascularity changes^9^, mapping pathologic variations^13,14^, or quantifying cancer stem cells^15^, and many other approaches^16–18^. However, targeting molecular mechanism of RT response for therapeutic prognosis is still rare. Inflammation is an essential mechanism of innate immune reactions responding to acute stimulations from microbial invasions or tissue injury. The hallmark of sterile inflammation is the rapid infiltration of polymorphonuclear neutrophils (PMNs), which can produce large amounts of reactive oxygen species (ROS) through the secretion of bactericidal and tumoricidal enzymes myeloperoxidase (MPO). Recent studies showed that exogenous ROS generation could lead to polarization of antitumor M1 tumor-associated macrophages and recruitment of cytotoxic lymphocytes and T cells^19,20^. It is worth noting that PMNs infiltration during an acute inflammation can augment the local ROS level by up to 20-fold^21,22^, in which the ROS itself can be a lethal factor to tumor cells^23^. More importantly, therapy-induced inflammatory responses are responsible to the adaptive immune responses through ROS-mediated cell apoptosis and the ensuing activation of immune T cells^24^, in which neutrophils may play an important role in bridging the innate and adaptive immunity. Taken together, we hypothesized that the ROS generation from radiation-induced acute inflammation may serve as a molecular target for early stratification of the RT outcomes in cancer therapy. Recently, ROS-based imaging probes have been extensively studied by implicating positron emission tomography (PET) and optical methods for assessing the disease states^25–29^. Although PET and optical methods can provide exclusive detection sensitivity, the lack of anatomical soft tissue contrast may limit the resolution of spatial distribution in tumor. This leads to a major gap to resolve the imaging results for accurate quantification and stratification. Magnetic resonance imaging (MRI) can provide excellent anatomical accuracy in soft tissues; however, conventional MRI fails to meet the criteria of high sensitivity and the approach to RT response stratification remains elusive.

**Fig. 1:**
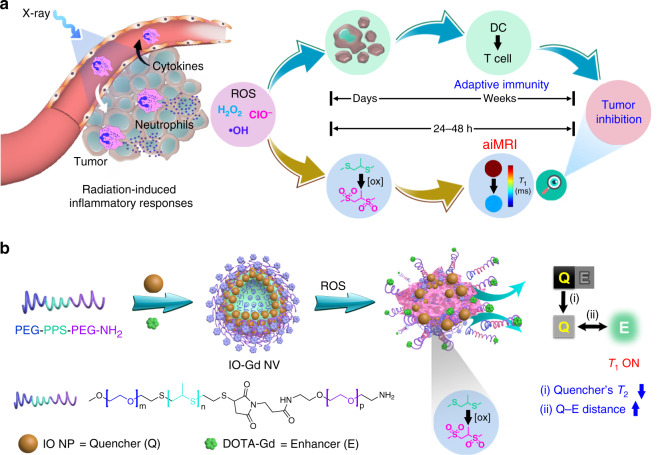
Illustration of the activatable inflammation magnetic resonance imaging (aiMRI). **a** Radiotherapy (RT)-induced acute inflammatory response leads to reactive oxygen species (ROS) production, which exerts tumor inhibition through ROS-induced cell apoptosis and T-cell activation pathways. The adaptive immune responses usually take days to weeks after RT. The aiMRI is applied to quantify the ROS at an early time (24–48 h) after RT which is proposed to stratify the tumor inhibition. **b** The procedure of self-assembly and disassembly of the aiMRI nanoprobe that is composed of iron oxide nanoparticles (IO NPs), gadolinium (Gd) species, and triblock PEG-PPS-PEG-NH_2_ polymers, denoted as IO-Gd NVs. The oxidation of hydrophobic thioethers to hydrophilic sulfones leads to swelling of the polymers and decomposition of the IO-Gd NVs. This procedure confers dual-positive factors to the *T*_1_ MRI OFF–ON effect: the quencher’s *T*_2_ effect is decreased upon disassembly due to the dispersed magnetic field coupling effect; (ii) the quencher-enhancer (Q-E) distance is increased due to the oxidation-induced swelling of polymers equipped with Gd species.

In this work, we proposed an activatable inflammation MRI (aiMRI) approach for early stratification of RT response in a quantitative manner (Fig. 1a). The radiation-induced inflammatory response is responsible for the infiltration of neutrophils and the production of ROS. On the one hand, the ROS-mediated cell apoptosis could lead to adaptive immune responses, which may take days to weeks after RT treatment. On the other hand, the inflammatory ROS can specifically oxidize hydrophobic thioethers to hydrophilic sulfones^30^, which can be engineered as a ROS-responsive platform for activatable MRI. The quantitative MRI is operated at 24–48 h after RT and is further examined by correlating the *T*_1_ relaxation changes with the corresponding tumor inhibition rates. The OFF–ON phenomenon for activatable *T*_1_ MRI is leveraged by the interplay between *T*_2_ quencher (Q) and *T*_1_ enhancer (E), which is mainly related to the quencher’s *T*_2_ effect and the Q–E distance. Here, we used triblock poly(ethylene glycol)–poly(propylene sulfide)–poly(ethylene glycol) (PEG–PPS–PEG) amphiphilic copolymers to fabricate a nanovesicle (NV) structure containing small-sized iron oxide nanoparticles (IO NPs) in the membrane and gadolinium (Gd) species on the surface, denoted as IO-Gd NVs (Fig. 1b). The self-assembly and disassembly of the IO-Gd NVs confer dual-positive factors to the *T*_1_ OFF–ON effect: (1) the quencher’s *T*_2_ effect is decreased upon disassembly due to the dispersed magnetic field coupling effect^31^; (2) the Q–E distance is increased due to the oxidation-induced swelling of polymers equipped with Gd species^32,33^. To facilitate quantification, we used MRI relaxation maps to quantify the *T*_1_ relaxation time changes in the aiMRI, allowing us to correlate the *T*_1_ relaxation changes derived from the aiMRI with the ensuing adaptive immune responses and the tumor growth rates in different treatment models.

## Results

### The aiMRI nanoprobe

**Fig. 2:**
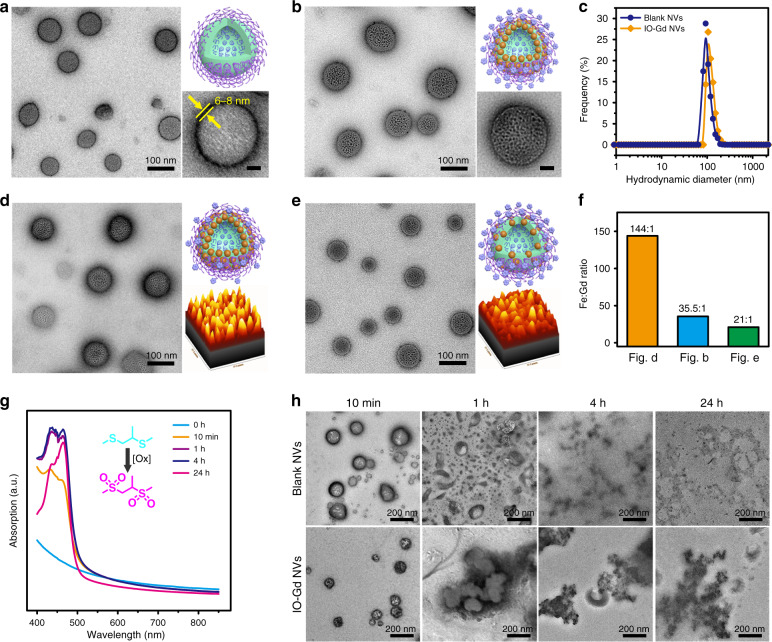
Characterizations of ROS-responsive IO-Gd NVs. **a** TEM image, cartoon image (upper right), and magnified TEM image (lower right, scale bar 20 nm) of the blank NVs. The membrane thickness of the blank NVs is about 6–8 nm. **b** TEM image, cartoon image (upper right), and magnified TEM image (lower right, scale bar 20 nm) of the IO-Gd NVs (Fe:Gd = 35.5:1). **c** DLS measurements of the blank NVs and the IO-Gd NVs. **d**–**f** TEM images and column analysis of the IO-Gd NVs with different Fe:Gd molar ratios. Insets show the cartoon figure and intensity-based analysis of the distribution of IO NPs according to the TEM image, respectively. **g** Time-dependent UV–vis absorption of the blank NVs incubated with NaCl, MPO (5 U/mL), and H_2_O_2_ (500 μM). **h** TEM images of the blank NVs and the IO-Gd NVs incubated with NaCl, MPO (5 U/mL), and H_2_O_2_ (500 μM) at different time points.

The triblock PEG–-PPS–PEG–NH_2_ copolymers were synthesized through a modified procedure from literature and characterized by proton nuclear magnetic resonance (NMR) spectrum (Supplementary Figs. 1−3). Self-assembly of the triblock copolymers led to the formation of blank NVs with diameters at around 100 nm from the transmission electron microscopy (TEM) images (Fig. 2a). The presence of amine terminal groups on the blank NVs was confirmed by the zeta-potential analysis (Supplementary Fig. 4). The membrane thickness of the black NVs is about 6–8 nm, indicating a uniform lamellar assembly structure. Previously, we have shown that small-sized hydrophobic Au NPs were able to incorporate into the membrane during the assembly of PPS–PEG NVs^30^. Here, we used 5 nm sized IO NPs as building-blocks to attain IO NVs which was further modified with Gd species to attain IO-Gd NVs (Fig. 2b and Supplementary Fig. 5). We further prepared single-component IO NVs and Gd NVs, which show similar hydrodynamic diameters with those of blank NVs and IO-Gd NVs (Fig. 2c and Supplementary Fig. 6). The IO-Gd NVs with different Fe:Gd ratios were obtained by tuning the feeding amount of IO NPs and Gd species (Fig. 2d–f). The three IO-Gd NVs with Fe:Gd ratios of 35.5:1, 144:1 and 21:1 for Figs. 2b, d, and e, respectively, show good uniformity both in size and morphology.

To investigate the degradation behavior of the blank NVs in response to an inflammatory milieu, we used H_2_O_2_ and MPO to mimic the inflammation-mediated oxidative burst in which MPO can convert H_2_O_2_ into HClO in the presence of chloride ions. While the oxidation strength of H_2_O_2_ is slightly higher than that of HClO^34^, the later has greater oxidation kinetics, which has been reported as a promising alternative to H_2_O_2_ with greatly enhanced oxidative efficiency^35^. We monitored the UV–vis absorption of the blank NVs at different time points after incubated with H_2_O_2_ (500 μM), MPO (5 U/mL), and NaCl (500 mM). The results show that the absorption between 400 and 475 nm had a remarkable increase at as early as 10 min after incubation, which may be ascribed to the formation of hypochlorites (Fig. 2g). Meanwhile, the gradually decreased intensity after 500 nm indicates the decomposition of the blank NVs due to the oxidation and swelling of the membrane. This phenomenon is comparable to the oxidation behavior of blank NVs incubated at a high concentration of H_2_O_2_ (10 mM) but without MPO (Supplementary Fig. 7A). On the contrary, the blank NVs show negligible changes in the absorption intensity incubated with H_2_O_2_ at 500 μM but without MPO (Supplementary Fig. 7B). The changes of chemical shifts in the proton NMR spectrum also confirmed the successful oxidation of PPS backbone (Supplementary Fig. 8). These results indicate that MPO can potentiate the efficacy of H_2_O_2_ to oxidize PPS units at an inflammation-relevant concentration. We further used TEM images to characterize the morphology changes of the blank NVs and IO-Gd NVs after inflammatory oxidation. By taking aliquots from the solutions at different time points, we observed a gradual degradation of the blank NVs initiated at around 10 min after incubation (Fig. 2h). The oxidation of hydrophobic PPS units leads to swelling of the membrane backbone of NVs, whereas the residual hydrophobic components underwent reverse micellation within 4 h and lasted to 24 h incubation time from the TEM images. This process was also monitored by DLS measurements which show gradually decreased hydrodynamic diameters with increasing the incubation time (Supplementary Fig. 9). For IO-Gd NVs, the DLS results show a slightly increase of hydrodynamic diameters at 10 min and 1 h incubation time probably due to the micellation of the residual IO NPs. At later time points of incubation, precipitation was observed in the IO-Gd NVs which further indicated the decomposition of the vesicular structures under the artificial inflammatory milieu.

**Fig. 3:**
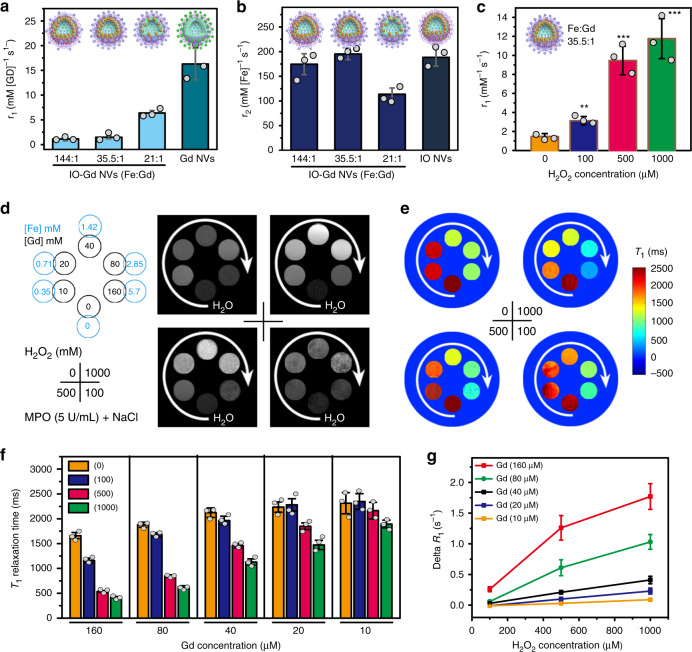
MRI measurements of the IO-Gd NVs. **a**, **b** The *r*_1_ and *r*_2_ values of the IO-Gd NVs with different Fe:Gd ratios, compared with Gd NVs and IO NVs, respectively. The *r*_1_ and *r*_2_ values were calculated on the basis of Gd and Fe elements for the IO-Gd samples, respectively. **c**, **d** The *r*_1_ values and *T*_1_ phantom images of the IO-Gd NVs (Fe:Gd = 35.5:1) incubated with different concentrations of H_2_O_2_ (0, 100, 500, and 1000 μM, lower right) in the presence of same amount of NaCl and MPO (5 U/mL). White arrows indicate the sample concentrations from low to high (d, upper left). MRI phantom acquisition parameters are TR = 500 ms and TE = 12.5 ms. **P = 0.0016; ***P < 0.001 (*n* = 3 independent samples, one-tailed paired *t*-test). **e**, The quantitative *T*_1_ relaxation time maps reconstructed from multiple *T*_1_ phantom images with TR = 50, 250, 500, 1000, 2000, 4000, 6000 ms, and TE = 12.5 ms. **f** Columns show the *T*_1_ relaxation times of the IO-Gd NVs at different conditions (*n* = 3 independent samples). **g** Analysis of the ∆*R*_1_ (1/*T*_1_) of the IO-Gd NVs at different conditions (*n* = 3 independent samples). All error bars indicate mean ± s.d.

The MRI performance of the IO-Gd NVs was evaluated on a 7 T MRI scanner. The *r*_1_ relaxivity values are inversely proportional to the Fe:Gd ratio of different IO-Gd NVs, from 1.13 ± 0.36 to 1.47 ± 0.31 and 6.39 ± 0.49 mM^−1^ s^−1^ for Fe:Gd ratios of 144:1 to 35.5:1 and 21:1, respectively (Fig. 3a). The sharp decrease of the *r*_1_ values from 6.39 to 1.47 mM^−1^ s^−1^ could be due to emerging magnetic field coupling effect, which is highly dependent on the distance between IO NPs. Interestingly, the *r*_2_ relaxivity values of two IO-Gd NVs (Fe:Gd ratios of 144:1 and 35.5:1) and the IO NVs are similar, 174.5 ± 21.3, 195.3 ± 11.5, and 188.4 ± 17.6 mM^−1^ s^−1^, respectively, which are much higher than that of the IO-Gd NVs with Fe:Gd ratio of 21:1 (*r*_2_ = 113.7 ± 12.7 mM^−1^ s^−1^) (Fig. 3b). Compared with the *r*_1_ value of the Gd NVs (16.3 ± 3.2 mM^−1^ s^−1^), these results indicate that the clustering effect of IO NPs could ‘quench’ the *T*_1_ relaxivity of adjacent Gd species due to the significantly enhanced *T*_2_ shortening effect of IO-Gd NVs. The *r*_2_ values of the single IO NPs and the Gd NVs are 63.5 ± 4.6 and 31.2 ± 1.5 mM^−1^ s^−1^ (Supplementary Table 1), respectively, which indicate that the *r*_2_ values of IO-Gd NVs are mainly due to the clustering effect of IO NPs. To investigate the MRI performance of the IO-Gd NVs in response to inflammation, we studied the *r*_1_ values of the IO-Gd NVs with Fe:Gd ratio of 35.5:1 considering the interplay between the *r*_1_ and *r*_2_ values. We used MPO (5 U/mL), NaCl (500 mM), and H_2_O_2_ of different concentrations (0, 100, 500, and 1000 μM) to mimic an inflammatory milieu for studying the aiMRI of the IO-Gd NVs. Four IO-Gd samples were prepared in the presence of different H_2_O_2_ concentrations and with different Gd and Fe concentrations. The *r*_1_ values of the IO-Gd NVs gradually increased by increasing the supplied amount of H_2_O_2_, 1.47 ± 0.31, 3.13 ± 0.44, 9.46 ± 1.5, and 11.76 ± 2.1 mM^−1^ s^−1^ for samples incubated with 0, 100, 500, and 1000 μM of H_2_O_2_, respectively (Fig. 3c and Supplementary Table 2, **P = 0.0016, ***P < 0.001; one-tailed paired *t*-test). These results indicate that MPO and NaCl alone do not cause the change of *r*_1_ value of IO-Gd NVs, instead, MPO and NaCl together are able to convert H_2_O_2_ into highly oxidative HClO. At a typical tumor concentration of 50 μM of H_2_O_2_, the changes of *r*_1_ values of the IO-Gd NVs were minimal either with or without the presence of MPO and NaCl (Supplementary Fig. 10). Moreover, the *r*_1_ values were also positively correlated with the MPO concentration under the same concentration of 500 μM of H_2_O_2_ (Supplementary Fig. 11). The *r*_2_ values of the IO-Gd NVs slightly decreased after oxidation due to the reverse micellation of the IO NPs (Supplementary Fig. 12 and Table 2). The *r*_1_ and *r*_2_ values of the Gd NVs show similar trends upon oxidation (Supplementary Fig. 13 and Table 2), with *r*_1_ value from 16.3 ± 3.2 to 10.2 ± 2.7 mM^−1^ s^−1^ and *r*_2_ value from 31.2 ± 1.5 to 19.5 ± 4.3 mM^−1^ s^−1^, which are consistent with the *T*_1_ phantom images. Similarly, the *r*_2_ value of the IO NVs dropped from 188.4 ± 17.6 to 117.5 ± 21.6 mM^−1^ s^−1^ after oxidation, while the *r*_1_ values had little change from 1.2 ± 0.4 to 1.4 ± 0.2 mM^−1^ s^−1^. The decreased *r*_1_ and *r*_2_ values for those samples can be attributed to the changes in the tumbling time of Gd species and the magnetic field coupling of IO NPs, respectively.

For the *T*_1_-weighted MRI phantoms, the IO-Gd NVs alone (0 μM of H_2_O_2_) show negligible *T*_1_ contrast between different concentrations (Fig. 3d). This phenomenon is due to the equipped mechanism of acquiring *T*_1_-weighted MRI that the longitudinal (*T*_1_) magnetization has to flip down to the transverse plane to be detected, giving rise to the ‘quenching’ phenomenon of *T*_2_ shortening effect to *T*_1_ contrast. In this respect, strong *T*_2_ effect may disparage the recovery of *T*_1_ magnetization during the *T*_1_ signal acquisition, reducing the *T*_1_ contrast and vice versa. Upon oxidation, the *T*_1_ bright contrast of the IO-Gd NVs appeared with increasing concentration of H_2_O_2_ while keeping the amount of NaCl and MPO constant, which may be attributed to the ROS-responsive decomposition of IO-Gd NVs and the swelling of Gd species. The *T*_1_ relaxation time maps were further constructed from a series of *T*_1_ phantoms of different parameters, allowing us to compare the relaxation changes in a quantitative manner (Fig. 3e, f). Moreover, the quantitative analysis of relaxation changes (∆*R*_1_ = 1/*T*_1, post_ – 1/*T*_1, pre_) indicates nearly linear correlations with the concentration of H_2_O_2_ (Fig. 3g).

### In vitro and in vivo evaluation of aiMRI

Cell viability assay shows that the blank and IO-Gd NVs have negligible cytotoxicity to both U87MG and 4T1 cells at polymer concentrations up to 800 μg/mL (Supplementary Fig. 14), indicating the good biocompatibility of these samples. To evaluate the feasibility of aiMRI in vivo, we established the mouse sterile inflammation model and conducted the *T*_1_ MRI using IO-Gd NVs, IO NVs (always off), and Gd NVs (always on) as contrast agents, respectively. The bright contrasts were observed in the inflammation spot for mice intravenously injected with IO-Gd NVs and Gd NVs with equivalent doses, whereas mice treated with IO NVs showed negative contrast in *T*_1_ MRI at 24 h post injection (p.i.) due to the dominant *T*_2_ effect (Supplementary Fig. 15A). The semi-quantitative signal-to-noise ratio (SNR) and contrast-to-noise ratio (CNR) analyses show that aiMRI using IO-Gd NVs could provide 2.4- and 9.1-fold enhancement for SNR and CNR of mouse inflammatory foci at 24 h p.i., respectively (Supplementary Fig. 15B). These results indicate that the IO-Gd NVs is a good candidate for aiMRI in vivo in terms of high biosafety and output efficiency.

**Fig. 4:**
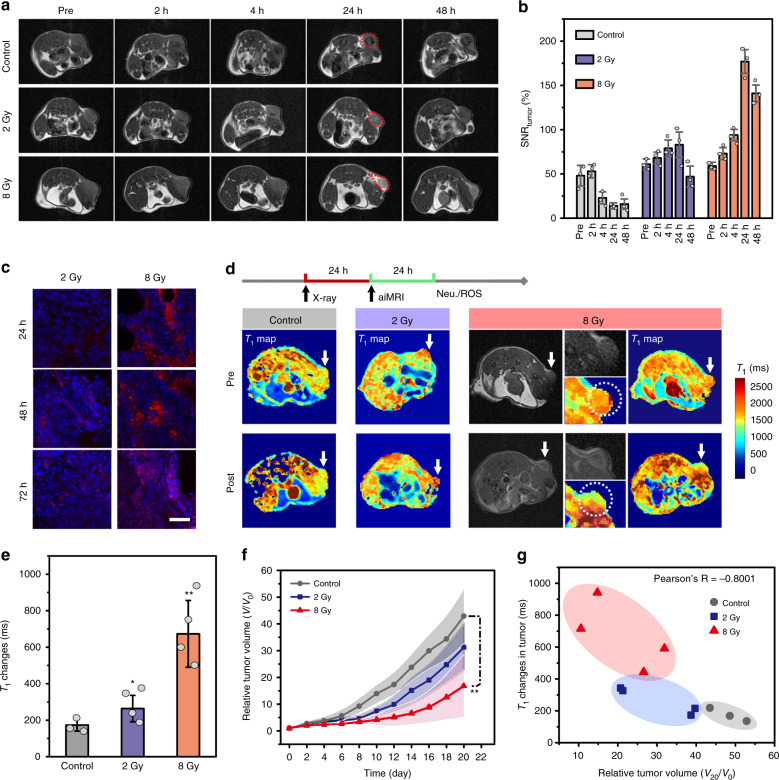
The aiMRI in mouse U87MG tumor models. **a** The *T*_1_ MRI study of mouse tumors after receiving 2 or 8 Gy X-ray irradiation and control, using IO-Gd NVs as contrast agents. The *T*_1_ MRI at 2, 4, 24, and 48 h post injection (p.i.) were acquired. Red dashed circles indicate the sharp contrast in tumors at 24 h p.i. **b** Semi-quantitative analysis of the signal-to-noise ratio (SNR) in tumors. **c** Immunofluorescence staining of the MPO levels in tumors at different time points after receiving X-ray irradiation (*n* = 3 biologically independent mice). **d** The pre- and post-contrast *T*_1_ phantom images and the quantitative *T*_1_ relaxation maps of mouse tumors. Radiotherapy (RT) was performed 24 h prior to the aiMRI acquisition. White arrows indicate mouse tumors. Magnified images indicate the tumor (white dotted circle). **e** Quantitative *T*_1_ relaxation time changes in mouse tumors. *P = 0.031 and **P = 0.0054 (*n* = 3 or 4 biologically independent mice indicated as dot plots, one-tailed homoscedastic *t*-test). **f** Tumor growth curves of the mouse groups (*n* = 5 biologically independent mice, **P = 0.0032, one-tailed paired t-test). The shadow indicates the errors. **g** Plotting of the *T*_1_ relaxation time changes at 24–48 h and the relative tumor volume changes at day 20 after RT. The Pearson’s correlation coefficient *R* is calculated to be −0.8001. All error bars indicate mean ± s.d.

To explore the aiMRI in mouse tumor after X-ray irradiation, we performed *T*_1_ MRI at subcutaneous U87MG tumors after receiving control (no irradiation), 2 or 8 Gy irradiation using IO-Gd NVs as contrast agents. The results show that the intravenously injected IO-Gd NVs turned into negative contrast in mouse tumors without irradiation (Fig. 4a). The accumulation of IO-Gd NVs led to gradually increased negative contrast in tumor with a maximum reached at 24 h p.i. The semi-quantitative analysis also reveals that the SNR_tumor_ decreases to a factor of 3.4 at 24 h p.i. compared with that of pre-contrast images (Fig. 4b). In contrast, mouse tumors receiving 8 Gy X-ray irradiation show bright contrast at 24 h p.i. with SNR_tumor_ of 177%, which slightly decreases to 141% at 48 h p.i. (Fig. 4a, b). The slightly decreased SNR_tumor_ is possibly due to the diffusion of Gd species after the decomposition of the IO-Gd NVs. However, mouse tumors receiving a low dose X-ray irradiation (2 Gy) show little *T*_1_ MRI signal changes and SNR_tumor_. The use of Gd NVs and IO NVs as contrast agents resulted in always ON and always OFF phenomenon in *T*_1_ MRI of mouse tumors receiving 8 Gy X-ray irradiation, which are consistent with the aiMRI in vitro and in vivo mouse inflammation models (Supplementary Fig. 16). We further explored the differences of inflammatory responses between mouse tumors receiving different doses of irradiation. The ELISA measurements of IL-6 and TNF-α show that the mouse group receiving 8 Gy irradiation produced significantly higher level of both inflammatory factors in the plasma compared with those of mouse group with 2 Gy irradiation, especially at 48 and 72 h post treatment time points (Supplementary Fig. 17A, B). This trend is also consistent with the IL-6 levels measured in the tumor mass, however, the differences of TNF-α level in the tumor mass between mouse groups receiving 2 and 8 Gy irradiation are marginal (Supplementary Fig. 17C, D). This phenomenon may be associated with the dysfunctional ROS resolution and oxidative DNA damage in the tumor rafter X-ray irradiation^36,37^. The hallmark of tumor inflammation is the infiltration of neutrophils which in turn secret specific tumoricidal enzymes MPO. Immunofluorescence staining results show that 8 Gy irradiation can elicit significantly higher level of MPO in mouse tumors compared with that of control group and mouse group with 2 Gy irradiation (Fig. 4c and Supplementary Fig. 18). The infiltration of neutrophils from peripheral vessels to deep tumor tissue over time was also observed.

Furthermore, we investigated the relationships between the early-time inflammatory ROS level and the late-time RT outcomes in U87MG mouse tumor models. We used quantitative aiMRI to stratify the inflammatory ROS levels at 24–48 h post irradiation of mouse groups receiving 0 (control), 2 or 8 Gy irradiation. The pre- and post-contrast *T*_1_-mapping MRI were acquired on each mouse of the three groups at 24 and 48 h after RT treatment, respectively. The representative *T*_1_ relaxation time maps of the three groups at respective pre- and post-contrast time points are presented at Fig. 4d, which were obtained from the reconstruction of a series of *T*_1_ parameters (Supplementary Fig. 19). Moreover, the multi-slice acquisition of MRI enables an anatomical analysis of the *T*_1_ relaxation time changes covering the whole frame of tumor (Supplementary Figs. 20 and 21). These results allow us to record the quantitative *T*_1_ MRI changes in tumor. For example, mice receiving 8 Gy irradiation had a remarkably higher *T*_1_ relaxation time changes (673 ± 183 ms), compared with that of control (173 ± 33 ms) and 2 Gy irradiation (264 ± 72 ms) (Fig. 4e, *p = 0.031 and **p = 0.0054, one-tailed homoscedastic *t*-test). The mouse group receiving 8 Gy irradiation shows slightly higher tumor uptake of IO-Gd NVs than that of control and 2 Gy irradiation (Supplementary Fig. 22), which are possibly due to the enhanced vascular permeability in tumor during inflammatory response. This result further underlies that the prominent *T*_1_ relaxation time changes in mice receiving 8 Gy irradiation are mainly ascribed to the aiMRI rather than other factors.

The tumor growth curves after RT show that mouse group receiving 8 Gy irradiation had significantly lower average tumor growth rates compared with the control groups (Fig. 4f, **p = 0.0032, one-tailed paired t-test). However, the tumor growth rates for individual mouse show great variations for all the treatment groups due to the heterogeneous RT responses (Supplementary Fig. 23). The mouse body weight and the survival rate were recorded at 20 and 40 days post irradiation, respectively (Supplementary Fig. 24). The representative hematoxylin and eosin (H&E) staining and the terminal deoxynucleotidyl transferase dUTP nick end labeling (TUNEL) immunofluorescence staining results show higher level of cell death for 8 Gy treated mice compared with control groups (Supplementary Fig. 25). To investigate the correlations between different factors in the RT, we plotted the *T*_1_ relaxation time changes and the corresponding tumor volume changes for individual mouse of different groups (Fig. 4g). The results show that mouse groups receiving different doses of irradiation have convergent correlations between each other, which are consistent to the averaged tumor growth curves. More importantly, the Pearson’s correlation coefficient (*R* = −0.8001) obtained from the divergent analysis indicates that higher *T*_1_ relaxation time change in tumor after RT is strongly negatively correlated to the smaller tumor volume change of mouse at 20 days post treatment. The strong Pearson’s *R* was also recorded between the irradiation doses, the *T*_1_ relaxation changes and the tumor inhibition rates (Supplementary Fig. 26), indicating the great promise of using aiMRI to stratify the RT outcomes.

### Stratification of RT response by aiMRI

**Fig. 5:**
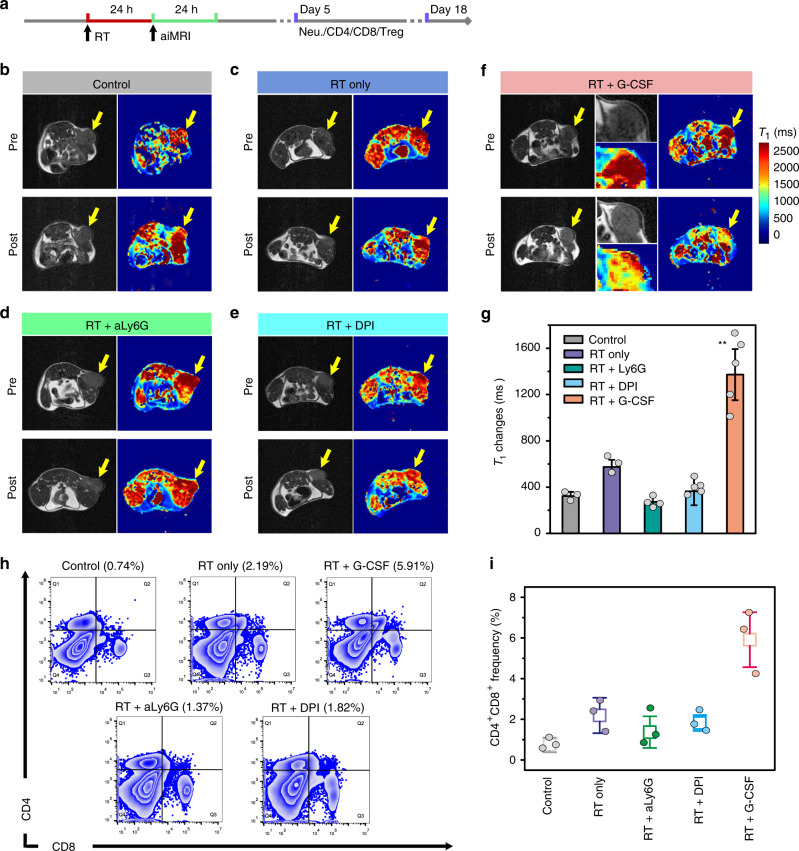
The aiMRI study in Balb/c mouse 4T1 tumor models. **a** Scheme shows the procedure time points of the radiotherapy (RT) study. **b**–**f** The pre- and post-contrast *T*_1_ phantom images and the quantitative *T*_1_ relaxation maps of mouse tumors after receiving control, RT (15 Gy) only, RT + aLy6G, RT + DPI, and RT + G-CSF, respectively. Yellow arrows indicate mouse tumors. The mouse tumor is magnified in the middle for the RT + G-CSF panel. **g** Quantitative *T*_1_ relaxation time changes in mouse tumors (*n* = 5 biologically independent mice, **P = 0.0014, one-tailed homoscedastic *t*-test). **h**, **i** Flow cytometry analysis and quantification of the CD4^+^CD8^+^ T cells in the splenocytes of mice at 5 days after different treatments (*n* = 3 biologically independent mice). All error bars indicate mean ± s.d.

Encouraged by the above results, we further investigated how aiMRI can be applied to bridge the innate and adaptive immunity after RT. We studied the aiMRI and the immune response in Balb/c mouse 4T1 tumor models. RT were conducted on four groups including RT only, RT + aLy6G (neutrophil depleting antibody), RT + G-CSF (granulocyte colony-stimulating factor), and RT + DPI (diphenyleneiodonium, a NADPH oxidase inhibitor). The RT dose of 15 Gy was chosen in the 4T1 tumor model due to the evidenced radiotherapy response^24^. Although the mechanism is not clear, it is accepted that a high dose ablative RT, rather than multiple low doses, is able to reduce the myeloid-derived suppressor cells, thus reversing the immune-suppressive nature in tumor^38,39^. We acquired the aiMRI for each mouse at pre- and post-contrast time points which corresponds to 24 and 48 h after RT treatment, respectively (Fig. 5a–f and Supplementary Fig. 27). The representative *T*_1_ mapping results shows that the control group received an averaged *T*_1_ relaxation time changes of 323 ± 37 ms (Fig. 5g, **P = 0.0014, one-tailed homoscedastic *t*-test). In addition, the mouse groups treated with RT + aLy6G and RT + DPI had much lower *T*_1_ relaxation time changes (273 ± 33 and 364 ± 121 ms, respectively) than that with RT only (574 ± 62 ms). In contrast, the highest *T*_1_ relaxation time changes were recorded in mice treated with RT + G-CSF (1372 ± 221 ms) according to the multi-slice analysis of the aiMRI results (Supplementary Figs. 28 and 29). This phenomenon can be ascribed to the enhanced CD11b^+^Gr-1^+^ neutrophil infiltration in tumor by RT and G-CSF stimulation (Supplementary Fig. 30). Moreover, the segregation of immature (CD101^−^) and mature (CD101^+^) neutrophil subsets revealed that mouse tumors treated with X-ray + G-CSF are responsible for a significantly higher fraction of mature neutrophils in the tumor, but not in the blood, when compared with the other groups (X-ray or G-CSF alone) (Supplementary Fig. 31). These results further indicate that RT-mediated neutrophil infiltration can contribute to the oxidative stress in tumor cells, which could be alternatively quantified by the aiMRI. At the fifth day after treatment, the ROS-mediated tumor cell death for mice treated with RT + G-CSF was 2.3-fold higher compared with that of RT only (Supplementary Fig. 32). Moreover, the frequency of CD4^+^CD8^+^ T cells in the splenocytes of mice examined at day 5 post treatment of RT + G-CSF was much higher (5.91%) than those of other groups ranging from 0.72% to 2.19% (Fig. 5h, i). In addition, the frequency of Treg cells in the splenocytes of mice treated with RT + G-CSF decreased to a lowest value of 0.67% among the other groups at day 5 post treatment, indicating the reversed immunosuppressive nature in tumor (Supplementary Fig. 33). This trend was further confirmed at the end point around day 18 after RT treatment, indicating the continuous immune responses throughout the posttreatment period (Supplementary Fig. 34).

**Fig. 6:**
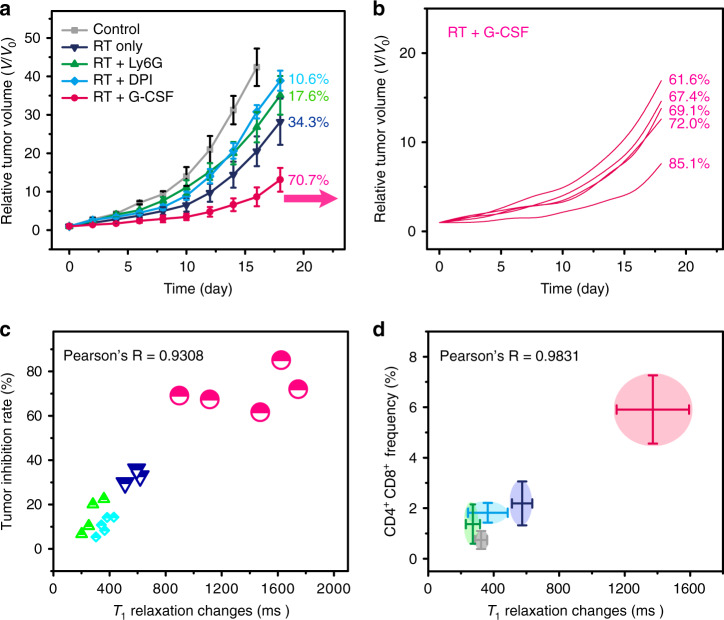
Stratification of the RT study in Balb/c mouse 4T1 tumor models. **a** The tumor growth curves of mouse groups (*n* = 5 biologically independent mice) with different treatments, including control, RT (15 Gy) only, RT + aLy6G, RT + DPI, and RT + G-CSF. The overall tumor inhibition rates are presented after each curve. **b** Individual tumor growth curves and the corresponding tumor inhibition rates for mouse treated with RT + G-CSF. **c** The correlations between the *T*_1_ relaxation time change and the corresponding tumor inhibition rate for individual mouse from different treatment groups except for the control. The Pearson’s correlation coefficient *R* is 0.9308. Mouse individuals are shown and mouse groups are indicated by different colors and symbols. **d** The correlations between the *T*_1_ relaxation time changes (with errors) and the CD4^+^CD8^+^ T cells (with errors) for mouse groups receiving different treatments. The Pearson’s correlation coefficient *R* is 0.9831. All error bars indicate mean ± s.d.

The tumor growth curves show that mice receiving RT + G-CSF treatment had a significantly lower growth rate and higher survival rate than other groups until 20 and 40 days after treatment, respectively (Fig. 6a and Supplementary Fig. 35). The tumor inhibition rate of the RT + G-CSF mouse group (70.7%) is about twofold, fourfold, and sevenfold higher than those of RT only (34.3%), RT + aLy6G (17.6%), and RT + DPI (10.6%) mouse groups, respectively. The individual mouse tumor inhibition rates were calculated to quantify the correlations between the tumor inhibition rates and the *T*_1_ relaxation time changes (Fig. 6b and Supplementary Fig. 36). The H&E staining results indicate negligible systemic toxicity to the major organs of mice in all treatment groups (Supplementary Fig. 37). The Pearson’s *R* value is 0.9308 derived from the correlations between the individual *T*_1_ relaxation time changes at 24–48 h and the corresponding growth inhibition rates at day 18 after RT treatments for all groups (Fig. 6c). This result indicates that the quantitative aiMRI approach is able to early stratify the efficacy of RT in the mouse model regardless the treatment methods. The high Pearson’s *R* value of 0.9831 is also obtained from the correlations between the averaged *T*_1_ relaxation time changes at 24–48 h and the frequency of CD4^+^CD8^+^ cells in splenocytes at day 5 after different treatments (Fig. 6d). Furthermore, we performed the aiMRI study in NADPH oxidase (Nox-2) deficient mice bearing B16F10 tumor (Supplementary Fig. 38). The results indicated that the Nox-deficient mice had significantly lower rate of inflammatory response derived from the aiMRI experiments, which correlated well with the lower tumor inhibition rate either with or without G-CSF treatment at late time (Pearson’s *R* = 0.9007).

## Discussion

We established our study on the hypothesis that acute inflammation-mediated oxidative burst may serve as a molecular mechanism for stratifying the therapeutic response in RT. It is known that inflammation can exert controversial effects on the malignant process with evidences for both pro-tumor and antitumor roles^40,41^. Recently, mounting evidence suggested that neutrophils alongside inflammation may have direct effect on regulating the malignant process of cancers^42–44^. One of the key features of neutrophil infiltration is the oxidative burst, which occurs concomitantly in tumor after exposure to radiation. It is important to point out that neutrophils contribute to the major source of MPO compared with monocyte-derived macrophages^45^, 1.8 mg versus 13 ng per 10^6^ neutrophils or macrophages, respectively. A recent work demonstrated that neutrophils play a key role in RT-induced antitumor effect in which the enhanced necrotic cell damage and tumor shrinkage were attributed to the ROS production^24^. The ensuing adaptive immunity after inflammatory ROS production was also confirmed^24^. Our study used aiMRI approach to quantify the RT-induced acute inflammatory ROS at 24–48 h post RT, which provided an insight of using ROS as a targeting mechanism for stratifying the RT response. Our results presented not only convergent correlations between enhanced ROS generation and improved tumor inhibition rate, but also divergent RT response in different individuals. These results further protrude the necessity of stratifying RT response at an early time for better management of cancer therapy.

MRI is a non-invasive and non-radiation method that is widely used in the clinic. The anatomical nature of MRI especially on soft tissues provides great opportunity in analyzing tissue structures and functions. Contrast agents are developed to enhance the contrast between imaging target and background in MRI, while activated MRI can further augment the sensitivity and specificity in diagnosis^46^. Designing *T*_1_-based MRI probes is usually achieved by sensing the distance between *T*_2_ quencher and *T*_1_ enhancer, in which a strong *T*_2_ quencher and a short Q–E distance result in efficient *T*_1_ signal quenching and vice versa^28,33^. In this study, we engineered both the *T*_2_ quencher effect and the Q–E distance to promote the sensitivity of the *T*_1_ OFF–ON phenomenon. The decomposition of IO-Gd NVs confers dual-positive factors to activate the *T*_1_ ON state of the conjugated Gd species: (1) small-sized IO NPs dismissed from the IO-Gd NVs significantly decrease the *T*_2_ quenching effect; (2) swelling of Gd species away from the IO NPs restores their intrinsic *T*_1_ effect. Upon stimulation, the Gd species are still conjugated on polymers which could benefit the *T*_1_ relaxivity due to the enhanced molecular tumbling time compared with that of unbounded Gd species generated by conventional cleavage strategy^47,48^. In this respect, we are able to use the aiMRI to stratify the ROS level within a biologically relevant range. On this occasion, the aiMRI may be amenable to imaging other inflammatory ROS-relevant diseases, such as atherosclerosis, arthritis, and sepsis. Last but not least, we used quantitative MRI to assess the *T*_1_ relaxation time changes due to the following reasons: (1) the relaxation changes can be correlated with the inflammatory ROS levels in a quantitative manner; (2) the radiologic technologists may benefit from the quantitative aiMRI during the radiation treatment planning (e.g., determining the delivery paths and dosages). Ultimately, successful treatment planning may lower the possibility of suffering from unnecessary side effects and, more importantly, lead to a higher likelihood of improved treatment outcomes. The situation of MPO deficiency in human needs to be carefully considered, which could result in low signal output in our approach. Therefore, further actions such as testing the MPO activity and modulating the therapeutic plan are needed for those cases.

To summarize, we developed an aiMRI strategy to early stratify the inflammatory ROS and the tumor inhibition rates in RT of different mouse models. The nanoprobe is equipped with dual-positive factors for sensing ROS with great sensitivity and specificity through activated *T*_1_ MRI in a quantitative manner. The *T*_1_ relaxation time changes at 24–48 h post RT show strong correlations with the ensuing adaptive immune responses and the tumor inhibition rates at the 18 days after RT, which provide an insight to early stratify RT response targeting the acute inflammatory ROS level. Moreover, the aiMRI approach may serve as a general tool to stratify the treatment efficacy of other ROS generating anticancer strategies. This study may shed new light on cancer diagnosis, prognosis and treatment planning for precision cancer therapy.

## Methods

### Synthesis of PEG–PPS–PEG amphiphilic triblock polymers

Poly(ethylene glycol) methyl ether (Mw 750) were used as source materials to obtain PEG-Tosyl and PEG thioacetate according to previously reported procedures. The PEG thioacetate were used to yield PEG–PPS–disulfide pyridine as follows: 200 mg of PEG thioacetate were dissolved in 4 mL of THF in a 10 mL Schlenk flask and degassed with N_2_. Sodium methoxide (16 mg, 0.5 M) were dissolved in 0.5 mL of MeOH and were then added to the flask through a syringe. After 30 min at room temperature, propylene sulfide (925 mg, 50 eq.) were injected to the system which was further degassed with N_2_ for three times. The system was left to react for 45 min and the end capping agent disulfide dipyridine (165 mg) was added and the system was left for 24 h. The product was precipitated at ethyl ether, washed with ethyl ether for three times and dried under vacuum overnight. The product PEG–PPS–disulfide pyridine (400 mg) were then dissolved in THF with the addition of thiol-poly(ethylene glycol)-amine (Mw 1k, 150 mg). The reaction was left for 48 h before adding ethyl ether for precipitation and washing for three times. The final product was dried under vacuum overnight and characterized by ^1^H NMR spectroscopy (Bruker, 300 MHz) in CDCl_3_.

### Preparation of nanovesicles

The PEG–PPS–PEG NVs were prepared using the solvent exchange method. Briefly, 6 mg of PEG–PPS–PEG were dissolved in 1.5 mL of THF. Deionized water was then added to the solution under vigorous stirring until the appearance of cloudy point. The solution was left open in fume hood until the completed evaporation of THF. The solution was then purified by low-speed centrifugation (3000 rpm) for 2 min to remove possible precipitation. A clear solution containing blank NVs was characterized by TEM and DLS measurements. The NVs equipped with IO NPs were obtained using a similar procedure by feeding as-synthesized oleic acid coated IO NPs (5 nm) during the formation of blank NVs. Furthermore, the amine terminated NVs then react with DOTA-NHS in borate buffer (pH = 8.5) for 2 h. Gd chloride of different ratios with respect to IO NPs were added to the solution at pH = 4.5 to yield IO-Gd NVs.

### MRI measurements

The MRI study was conducted on a 7 T scanner (Bruker). *T*_1_ MRI experiments were acquired using rapid acquisition with relaxation enhancement with variable repetition time (RARE-VTR) sequence. *T*_2_ MRI experiments were acquired using multi-slice multi-echo. The phantom samples with different concentrations of IO (Fe ions) and Gd were prepared in solution with addition of different amount of H_2_O_2_ and MPO as required. The *T*_1_ MRI phantom was acquired using the following parameters: echo time = 12.507 ms, effective TE = 12.507 ms, number of experiments = 7, multiple repetition time = 50, 250, 500, 1000, 2000, 4000, 6000 ms, flip angle = 180, rare factor = 2, number of repetitions = 1, number of averages = 2, matrix = 256 × 256. The *T*_2_ MRI phantom was acquired using the following parameters: echo time = 10 ms, effective TE = 10, 20, 30, 40, 50, 60, 70, 80, 90, 100, 110, 120, 130, 140, 150, 160 ms, number of repetitions = 1, flip angle = 180, repetition time = 2000 ms, number of averages = 2, matrix = 256 × 256.

### In vivo MRI of mouse tumor models

The in vivo MRI of subcutaneous tumors was conducted on a 7 T scanner (Bruker). All animal experiments were performed under the National Institutes of Health Clinical Center Animal Care and Use Committee (NIH CC/ACUC) approved protocol. The multi-slice *T*_1_ MRI was acquired with RARE-VTR sequence using the following parameters: echo time = 10.3 ms, effective TE = 10.3 ms, rare factor = 2, flip angle = 180, number of experiments = 1, repetition time = 350 ms, number of averages = 8, number of repetitions = 1, matrix = 256 × 256. The IO-Gd NVs were then injected intravenously with an injection dose of 10 μmol [Gd]/kg mouse body weight. The *T*_1_ mapping aiMRI experiments were acquired using RARE-VTR sequence with the following parameters: echo time = 10.3 ms, effective TE = 10.3 ms, rare factor = 2, flip angle = 180, number of experiments = 7, multiple repetition time = 6000, 4000, 2000, 1000, 500, 250, and 169.3 ms, number of averages = 2, number of repetitions = 1, Matrix = 128 × 128. For the aiMRI, the IO-Gd NVs were then injected intravenously with an injection dose of 4 μmol [Gd]/kg mouse body weight. The post-contrast MRI was acquired 24 h after the injection of contrast agents using the same sequence parameters as those for pre-contrast *T*_1_ MRI study.

### Study on the mouse U87MG tumor model

All animal experiments were performed under the National Institutes of Health Clinical Center Animal Care and Use Committee (NIH CC/ACUC) approved protocol. The U87MG cells mouse tumor model was established by subcutaneously injecting 2 × 10^6^ cells into the right back flank of mice (athymic nude, 5–6 weeks old). After the tumor size reached around 35–42 mm^3^, mice were randomly grouped into three groups (*n* = 5). The mouse groups were treated with different doses of X-ray irradiation at day 0 including 0 (control), 2, and 8 Gy. After 24 h, mice from different groups were scanned individually by MRI using *T*_1_ mapping sequence and multi-slice *T*_1_-weighted MRI sequence (pre-contrast MRI). The IO-Gd NVs were then injected intravenously with the injection dose of 4 μmol [Gd]/kg mouse body weight. At another 24 h after injection of contrast agents, the post-contrast MRI were acquired using the same *T*_1_ mapping sequence and multi-slice *T*_1_-weighted sequence as for pre-contrast MRI. The data were analyzed using NIH developed software ImageJ. The tumor size and body weight were recorded every 2 days after each treatment until 20 days post irradiation, and the tumor volumes were calculated by the equation: *V* = width^2^ × length/2. The survival rate was recorded for 40 days post irradiation.

### Study on the Balb/c mouse 4T1 tumor model

All animal experiments were performed under the National Institutes of Health Clinical Center Animal Care and Use Committee (NIH CC/ACUC) approved protocol. The 4T1 cells mouse tumor model was established by subcutaneously injecting 1 × 10^6^ cells into the right back flank of mice (athymic nude, 4–5 weeks old). After the tumor size reached around 50–60 mm^3^, mice were randomly grouped into five groups (*n* = 8 for each group, *n* = 5 for tumor growth monitoring and *n* = 3 for checking the immune responses). The mouse groups were treated at the day 0 including control group, RT only (15 Gy), RT + anti-Ly-6G (neutrophils depleting antibody, 200 μg per mouse, intraperitoneal injection at 24 h before RT), RT + G-CSF (granulocyte colony-stimulating factor, 3 μg per mouse, intradermal injection after RT, one time per day for four days), RT + DPI (diphenyleneiodonium, a NADPH oxidase inhibitor, 50 μg per mouse, intraperitoneal injection after RT).

After 24 h post RT, the aiMRI was conducted for mice from different groups (*n* = 5) using *T*_1_ mapping sequence and multi-slice *T*_1_-weighted MRI sequence (pre-contrast MRI). The IO-Gd NVs were then injected intravenously with the injection dose of 4 μmol [Gd]/kg mouse body weight. At another 24 h after injection of contrast agents, the post-contrast MRI experiments were acquired using the same sequences as for pre-contrast MRI. The data were analyzed using NIH developed software ImageJ. The tumor size and body weight were recorded every 2 days after each treatment until 18 days post irradiation, and the tumor volumes were calculated by the equation: *V* = width^2^ × length/2. The survival rate was recorded for 40 days post irradiation. The spleens and tumors from different groups (*n* = 3) were dissected at day 5 and the end point (day 18) after the X-ray irradiation. The isolated splenocytes and tumor cells were stained with different antibodies and the analysis was conducted by flow cytometry. Neutrophils from tumor cells were stained with APC anti-mouse Ly-6G/Ly-6C (Gr-1) and PE/Cy5 anti-mouse/human CD11b. Treg cells were stained following the protocol of the mouse Treg flow kit (FOXP3 Alexa Fluor 488/CD4 APC/CD25 (Biolegend).

### Reconstruction of the aiMRI maps

All the simulations were performed on home-written programs^49^ in MATLAB 2018a (MathWorks, Natick, MA, USA). In the phantom experiments where the signal in some voxels reached noise floor at large TEs, the signal correction for Rician noise was performed. The quantitative *T*_1_ maps were calculated from the multi-TR signals with non-linear least square fitting of the data with the following equation: $M\left( {{\mathrm{TR}}} \right) = M_0\left[ {1 - \exp \left( { - {\textstyle{{{\mathrm{TR}}} \over {T_1}}}} \right)} \right]$, where *M*(TR) was the signal intensity at each TR and *M*_0_ was a free fitting variable and equal to *M* = (TR = +inf).

### Statistical analysis

Student’s *t* tests were used for evaluating differences between groups. No samples were excluded from analysis except for specifically noted. Quantitative data are expressed as means ± s.d. (standard deviation). The statistical significance is indicated as *P < 0.05, **P < 0.01, and ***P < 0.001.

### Reporting summary

Further information on research design is available in the Nature Research Reporting Summary linked to this article.

## Supplementary information

Supplementary Information

Reporting Summary

## Data Availability

The authors declare that the data supporting the findings of this study are available within the article and its Supplementary Information Files or from the corresponding author upon reasonable request.
