# Supplementary material for: Early stratification of radiotherapy response by activatable inflammation magnetic resonance imaging
Source: Nat Commun. 2020 Jun 15;11:3032. doi: 10.1038/s41467-020-16771-y (PMC7295999; doi:10.1038/s41467-020-16771-y)
Supplement: Supplementary file 1 — Supplementary Information [file 41467_2020_16771_MOESM1_ESM.docx]

***Supplementary Information***

**Early stratification of radiotherapy response by activatable inflammation magnetic resonance imaging**

Zijian Zhou^1^, Hongzhang Deng^1,2^, Weijing Yang^1^, Zhantong Wang^1^, Lisen Lin^2^, Jeeva Munasinghe^3^, Orit Jacobson^1^, Yijing Liu^1^, Longguang Tang^1^, Qianqian Ni^1^, Fei Kang^1^, Yuan Liu^1^, Gang Niu^1^, Ruiliang Bai^4^, Chunqi Qian^5^, Jibin Song^2^*, Xiaoyuan Chen^1^*

^1^Laboratory of Molecular Imaging and Nanomedicine, National Institute of Biomedical Imaging and Bioengineering, National Institutes of Health, Bethesda, MD 20892, United States

^2^MOE Key Laboratory for Analytical Science of Food Safety and Biology, College of Chemistry, Fuzhou University, Fuzhou 350116, Fujian, China

^3^Laboratory of Functional and Molecular Imaging, National Institute of Neurological Disorders and Stroke, National Institutes of Health, Bethesda, Maryland 20892, United States

^4^Interdisciplinary Institute of Neuroscience and Technology, Qiushi Academy for Advanced Studies, Key Laboratory of Biomedical Engineering of Ministry of Education, College of Biomedical Engineering and Instrument Science, Zhejiang University, Hangzhou 310027, China

^5^Department of Radiology, Michigan State University, East Lansing, MI 48824, United States

Email: [shawn.chen@nih.gov](mailto:shawn.chen@nih.gov) (X. C.); [jibinsong@fzu.edu.cn](mailto:jibinsong@fzu.edu.cn) (J. S.)

This PDF file includes

**Supplementary Methods**………………………………………………………………………………S4

**Supplementary Fig. S1 | Synthetic route of the amphiphilic triblock PEG-PPS-PEG-NH_2_**………S7

**Supplementary Fig. S2 | ^1^H NMR spectrum of the PEG-PPS-disulfide pyridine polymers**………S8

**Supplementary Fig. S3 | ^1^H NMR spectrum of the PEG-PPS-PEG polymers**……………………..S9

**Supplementary Fig. S4 | Zeta potential analysis of the two nanovesicles**………………………….S10

**Supplementary Fig. S5 | TEM images of hydrophobic IO NPs of 5 nm in diameter**……………..S11

**Supplementary Fig. S6 | TEM and DLS measurements of the IO NVs and Gd NVs**…………….S12

**Supplementary Fig. S7 | Time-dependent UV-vis absorption of the blank NVs**………………….S13

**Supplementary Fig. S8 | The proton NMR spectrum of the triblock polymers after oxidation**…S14

**Supplementary Fig. S9 | DLS measurements of the blank NVs and IO-Gd NVs**…………………S15

**Supplementary Fig. S10 | The *r*_1_ values of the IO-Gd NVs (Fe:Gd = 35.5:1) at 50 µM of H_2_O_2_**…S16

**Supplementary Fig. S11 | The *r*_1_ values of the IO-Gd NVs at different MPO concentrations**…...S17

**Supplementary Fig. S12 | The *r*_2_ values of the IO-Gd NVs at different conditions**……………….S18

**Supplementary Fig. S13 | The *T*_1_ MRI study of the Gd NVs and IO NVs**…………………………S19

**Supplementary Fig. S14 | Biocompatibility study of the blank NVs and IO-Gd NVs**…………….S20

**Supplementary Fig. S15 | The aiMRI of inflammation in mouse muscle model**………………....S21

**Supplementary Fig. S16 | The aiMRI study of mouse tumor after X-ray irradiation**…………...S22

**Supplementary Fig. S17 | ELISA analysis of IL-6 and TNF-α in mouse plasma and tumor**…….S23

**Supplementary Fig. S18 | Immunofluorescence staining of MPO level in mouse tumor**…………S24

**Supplementary Fig. S19 | The multi-parametric *T*_1_ MR images**…………………………………...S25

**Supplementary Fig. S20 | Additional multi-parametric *T*_1_ MR images**…………………...………S26

**Supplementary Fig. S21 | Additional *T*_1_ MRI relaxation time maps**…..…………………...……..S27

**Supplementary Fig. S22 | Tumor accumulation measurements of the IO-Gd NVs**………………S28

**Supplementary Fig. S23 | Individual mouse tumor growth curves**..……………………….………S29

**Supplementary Fig. S24 | The mouse body weight and survival rate**……………………………...S30

**Supplementary Fig. S25 | The representative H&E and TUNEL staining results**..………………S31

**Supplementary Fig. S26 | The Pearson’s correlation analysis of RT in U87 MG tumor model**...S32

**Supplementary Fig. S27 | The multi-parametric *T*_1_ MR images of 4T1 tumors**……………...…...S33

**Supplementary Fig. S28 | The multi-parametric multi-slice *T*_1_ MR images**.……….………...…...S34

**Supplementary Fig. S29 | Additional multi-slice *T*_1_ MRI relaxation time maps**……………….…S35

**Supplementary Fig. S30 | Flow cytometry analysis of CD11b^+^Gr-1^+^ neutrophils in tumor**…..….S36

**Supplementary Fig. S31 | Flow cytometry analysis of immature and mature neutrophils after different treatments**………………………………………………………….……………………S37

**Supplementary Fig. S32 | Analysis of apoptotic tumor cell death**……………………………….....S38

**Supplementary Fig. S33 | Flow cytometry analysis of Treg cells in splenocytes**…………………..S39

**Supplementary Fig. S34 | Flow cytometry analysis of CD4^+^CD8^+^ T lymphocytes**………………..S40

**Supplementary Fig. S35 | The RT study in Balb/c mouse 4T1 tumor models**…………………….S41

**Supplementary Fig. S36 | Individual mouse 4T1 tumor growth curves**…………………………...S42

**Supplementary Fig. S37 | H&E staining of major organs after RT treatments**…………………..S43

**Supplementary Fig. S38 | The aiMRI study in Nox-2 deficient mouse models with B16F10 tumors**………………………………………………………………………………………………….S44

**Supplementary Table S1 | Summary of the *r*_1_ and *r*_2_ values of samples**…..…..…………..……….S46

**Supplementary Table S2 | Summary of the *r*_1_ and *r*_2_ values of samples after oxidation**………….S47

**Supplementary Methods**

**Materials.** Poly(ethylene glycol) methyl ether (Mw 750), p-toluenesulfonyl chloride (reagent grade, 98%), sodium methoxide (reagent grade, 95%), potassium thioacetate (98%), propylene sulfide (>96%), gadolinium chloride (99.9%), myeloperoxidase were purchased from Sigma-Aldrich. Thiol-poly(ethylene glycol)-amine (Mw 1k) were purchased from Creative PEGWorks. IL-6 and TNF-α (mouse), ELISA kits were purchased from Enzo Life Sciences. In situ cell death detection kit was purchased from Roche. Anti-myeloperoxidase antibody (Rabbit monoclonal EPR20257, ab208670) was purchased from Abcam. APC anti-mouse Ly-6G/Ly-6C (Gr-1), PE/Cy5 anti-mouse/human CD11b, mouse Treg flow kit (FOXP3 Alexa Fluor 488/CD4 APC/CD25 were purchased from Biolegend. Diphenyleneiodonium chloride was purchased from Alfa. Granulocyte Colony Stimulating Factor was purchased from GSS. All other products and solvents were purchased from Sigma without further purification.

C**haracterizations.** Transmission electron microscopy (TEM) images were acquired on a FEI Tecnai12 equipment with a voltage at 120 kV. UV-vis spectrum was recorded by a Genesys 10S UV-Vis spectrophotometer (Thermo Scientific, Waltham, MA) using quartz cuvettes with an optical path of 1 cm. Dynamic light scattering measurements were performed at a SZ-100 nano particle analyzer (HORIBA Scientific, USA). Proton nuclear magnetic resonance (^1^H NMR) spectra were recorded on a Bruker AV300 scanner. Inductively coupled plasma optical emission spectroscopy (ICP-OES) was used to quantify Fe and Gd concentrations. Athymic nude mice were purchased from Envigo (USA). Tissue and tumor samples for eosin (H&E) staining were prepared by BBC Biochemical (Mount Vernon, WA) and observed using a BX41 bright field microscopy (Olympus). Magnetic resonance imaging (MRI) measurements were performed on a Bruker 7 T scanner (Pharmascan) equipped with small animal-specific body coil or self-made mouse brain-specific coil. Two-tailed paired and unpaired Student’s t test was used to determine the significance between groups (*P < 0.05, **P < 0.01, ***P < 0.001). Data are presented as mean ± s.d.

**Synthesis of iron oxide nanoparticles.** To a 10 mL of benzyl ether solution, 353 mg of Fe(acac)_3_, 1.25 g of 1,2-hexadecanediol, 1 mL of oleic acid, 1 mL of oleyl amine were added in a three-neck round-bottom flask. The system was degassed with N_2_ at 120 °C for 20 min. Next, the flask was heated to 300 °C with a heating rate of 6-8 °C/min and maintained reflux for another 30 min. The system was then cooled to room temperature and the solution was added with ethyl alcohol to precipitate the nanoparticles. The products were collected by centrifugation at 7000 rpm for 12 min and further washed with hexane and ethyl alcohol for three times. Finally, the nanoparticles were dispersed in hexane and stored at 4 °C for further use.

**UV-vis measurements.** The blank PPS nanovesicles (5 mg/mL) were dissolved in a phosphate buffer (0.1 M) at pH 6.0. The UV-vis absorption of the blank NVs before oxidation was measured by Genesys 10S UV-Vis spectrophotometer (Thermo Scientific, Waltham, MA) at a wavelength range of 400 to 850 nm. The solution was then independently incubated with three different oxidation conditions: 0.5 mM H_2_O_2_, 0.5 mM H_2_O_2_ + MPO (5 U/mL) + NaCl, and 100 mM H_2_O_2_. The UV-vis absorption of these solutions was measured at 10 min, 1, 4, and 24 h after incubation. The reaction between MPO, H_2_O_2_ and NaCl yielded yellowish NaClO which show intense absorption at 410-480 nm. The UV-vis absorption of the blank NVs after 500 nm show slightly increase at early time and then decrease upon oxidation.

**Degradation of nanovesicles.** The degradation of blank and IO-Gd NVs was monitored by TEM analysis at different incubation time. Typically, the solution was incubated with H_2_O_2_ (0.5 mM), MPO (5 U/mL), and NaCl (500 mM) at pH = 6.0. The aliquots of solution after 10 min, 1, 4, and 24 h incubation were taken out to prepare the TEM samples. The TEM images were acquired on a FEI Tecnai12 equipment with a voltage at 120 kV.

**Cell viability assay.** The cell viability was evaluated on both U87MG and 4T1 cell models. Cells were first seeded in a 96-well plate at 1×10^4^ cells/well and incubated at 37 °C for 24 h. Blank NVs or IO-Gd NVs at different concentrations were added to each well (n = 3). Cells were then incubated for another 48 h before adding cell counting kit-8 (CCK-8). The cell viability for each concentration was derived from the Uv-vis absorption at 450 nm according to the following equation: [(Absorption of sample – absorption of background) / (absorption of control - absorption of background)] * 100%.

**Immunofluorescence staining of myeloperoxidase.** The tumor model were established by subcutaneously injecting U87 MG cells (4 × 10^6^) into the right back flank of mice (athymic nude, 5-6 weeks old). All animal experiments were performed under the National Institutes of Health Clinical Center Animal Care and Use Committee (NIH CC/ACUC) approved protocol. After the tumor size reached around 50 mm^3^, mice were randomly grouped into 7 groups (n = 3 per group). The mouse groups were subjected to receive X-ray irradiation (2 or 8 Gy, each with 3 groups) and a control group (no irradiation). The mouse groups were sacrificed at 24, 48, and 72 h after irradiation (both 2 and 8 Gy groups for each time point) and the plasma and the tumors were collected for Elisa and immunofluorescence staining according to manufacturer’s protocols. For immunofluorescence staining of myeloperoxidase, rabbit monoclonal anti-mouse myeloperoxidase antibody and cy3.5 conjugated anti-rabbit secondary antibody were used, and the slides were analyzed on a Zeiss LSM 780 confocal microscopy.


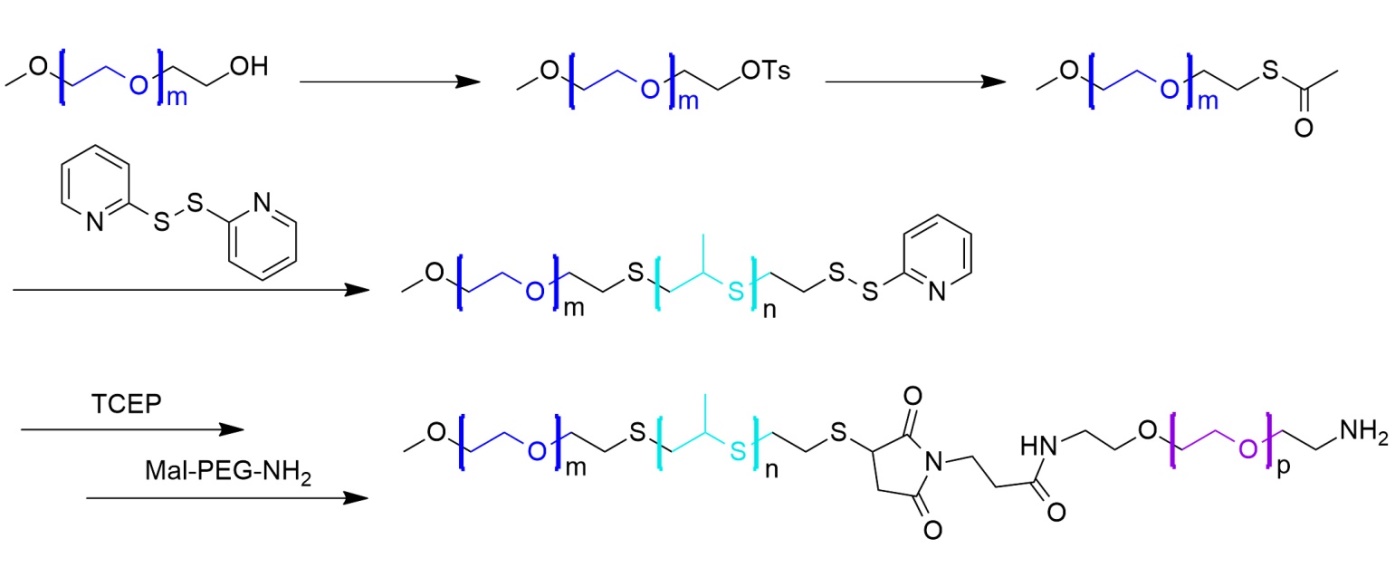


**Supplementary Fig. S1 | Synthetic route of the amphiphilic triblock PEG-PPS-PEG-NH_2_**. Methyl PEG_750_ (m = 16) is used as source material. OTs represents to tosylate group. TCEP represents to tris(2-carboxyethyl) phosphine. Mal-PEG_1000_-NH_2_ (p = 21) was used in the final step.


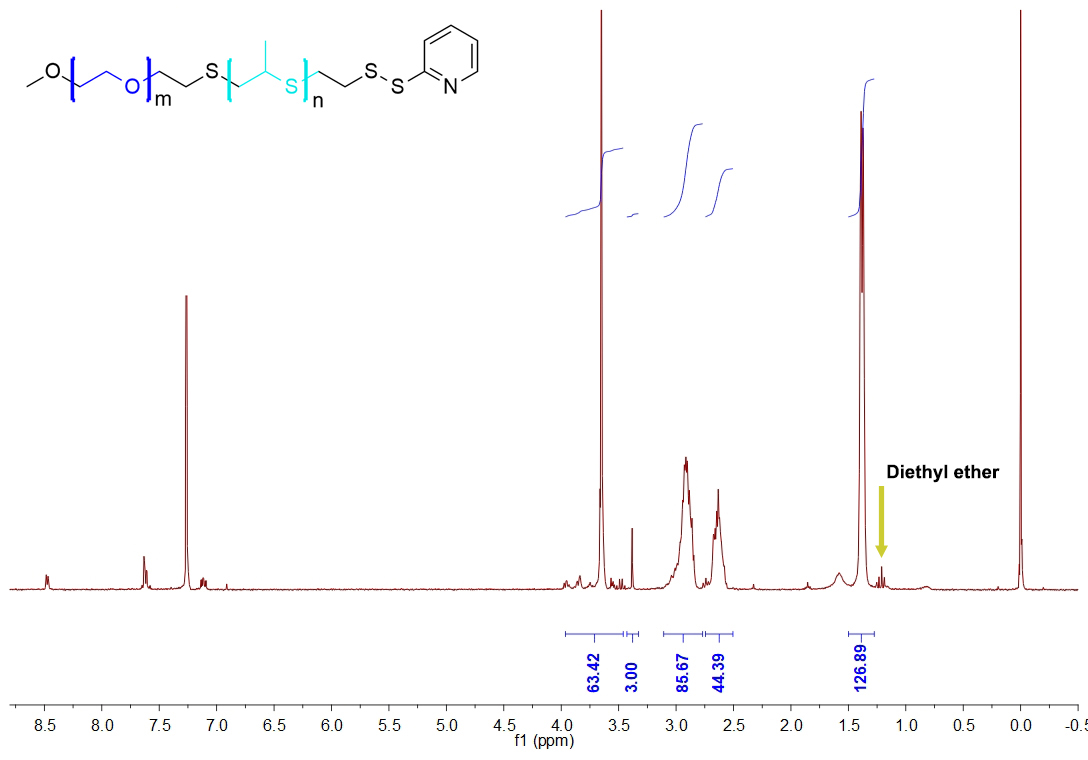


**Supplementary Fig. S2 | ^1^H NMR spectrum of the PEG-PPS-disulfide pyridine polymers.** The n = 44.39 is calculated from the integrals under the chemical shift at around 2.65 ppm. The impurity of diethyl ether at around 1.2 ppm is indicated. The NMR spectrum was recorded at 300 MHz.


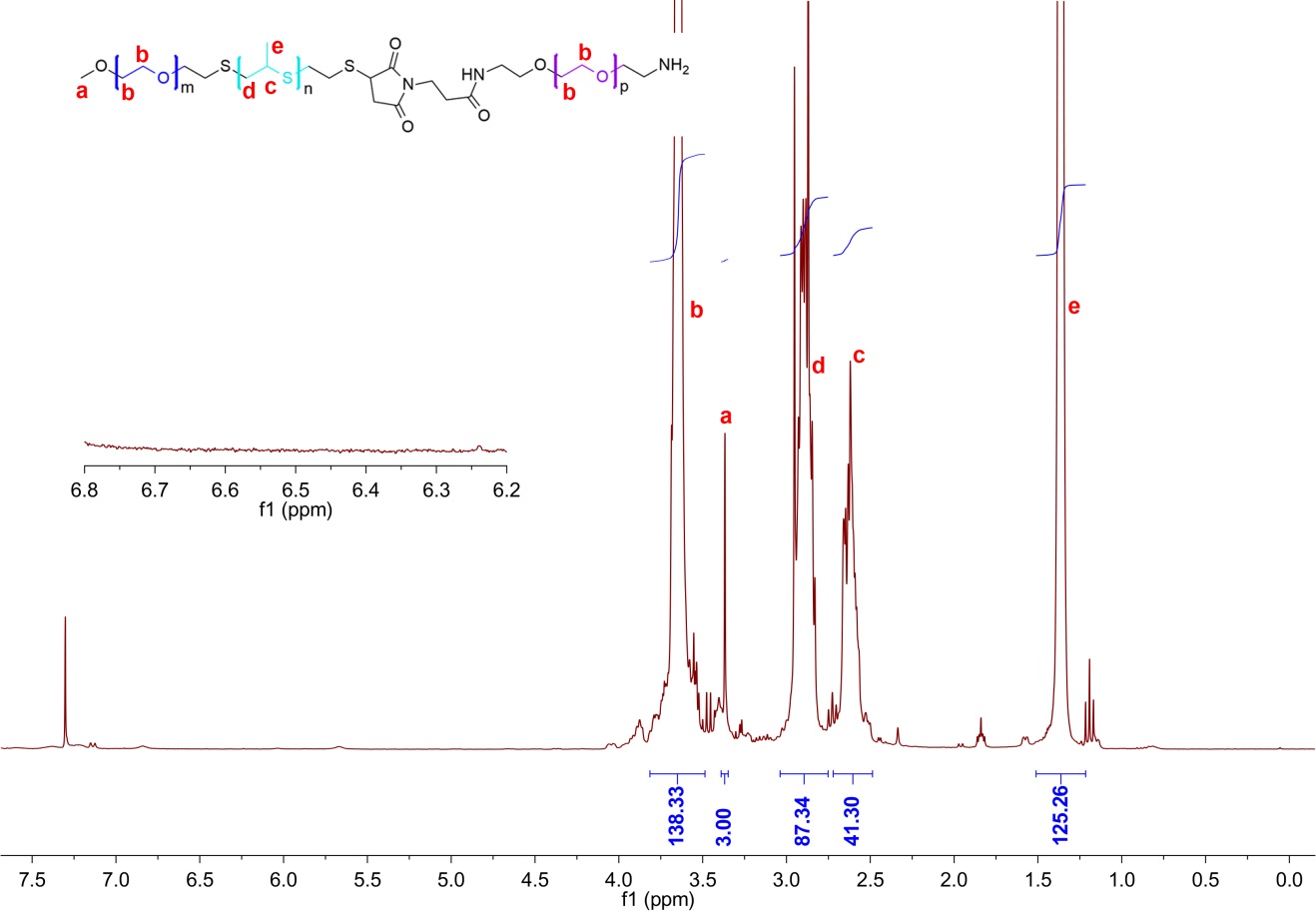


**Supplementary Fig. S3 | ^1^H NMR spectrum of the PEG-PPS-PEG polymers.** The chemical shift at around 1.2 ppm indicates impurity from diethyl ether. The NMR spectrum was recorded at 300 MHz.


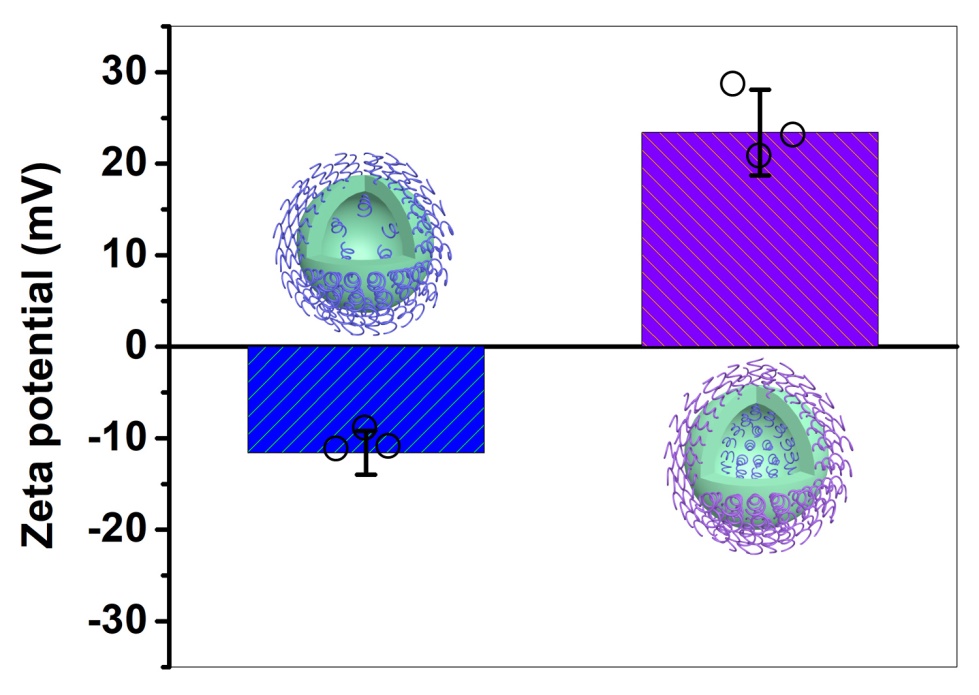


**Supplementary Fig. S4 | Zeta potential analysis of the two nanovesicles.** Self-assembly of the methyl PEG_750_-PPS-PEG_1000_-NH_2_ triblock copolymers leads to positive zeta potential due to the fact that the amine terminal ends are prone to stretch to the outer surface of the nanovesicles. In contrast, self-assembly of methyl PEG-PPS diblock copolymers leads to slightly negative zeta potential. Data represents mean ± s.d. (n = 3).


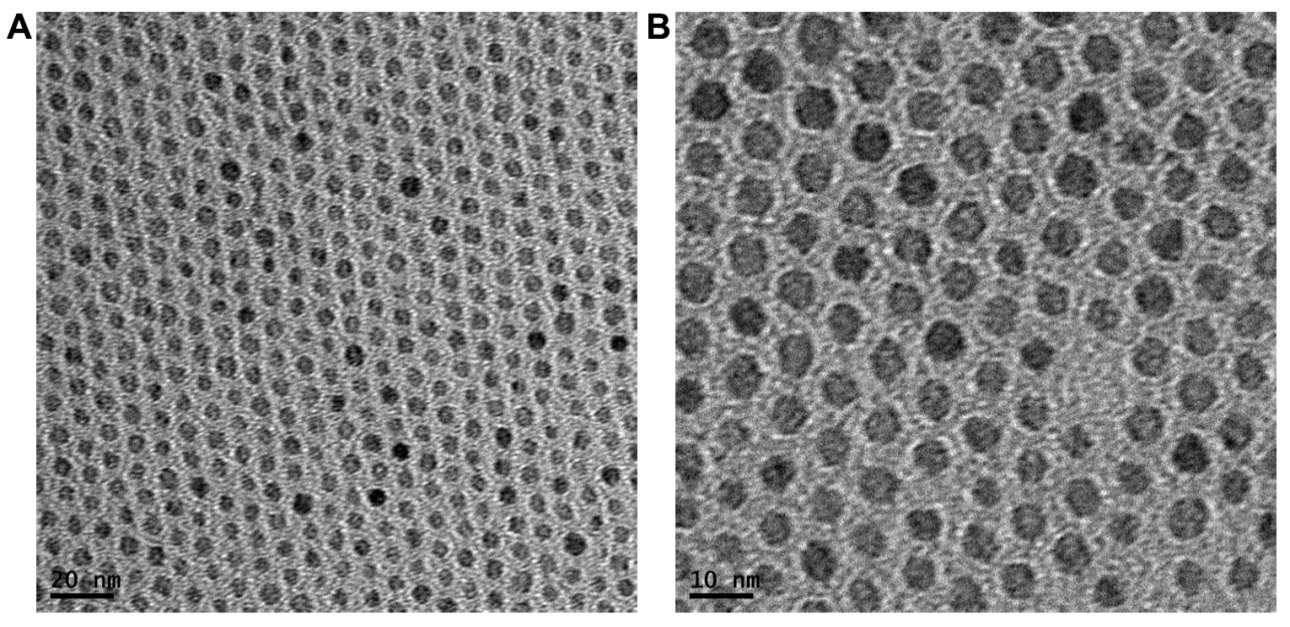


**Supplementary Fig. S5 | TEM images of hydrophobic IO NPs of 5 nm in diameter.** (A) scale bar 20 nm. (B) Magnified TEM image. Scale bar 10 nm. The as-synthesized IO NPs are coated with oleic acid and oleyl amine on the surface.


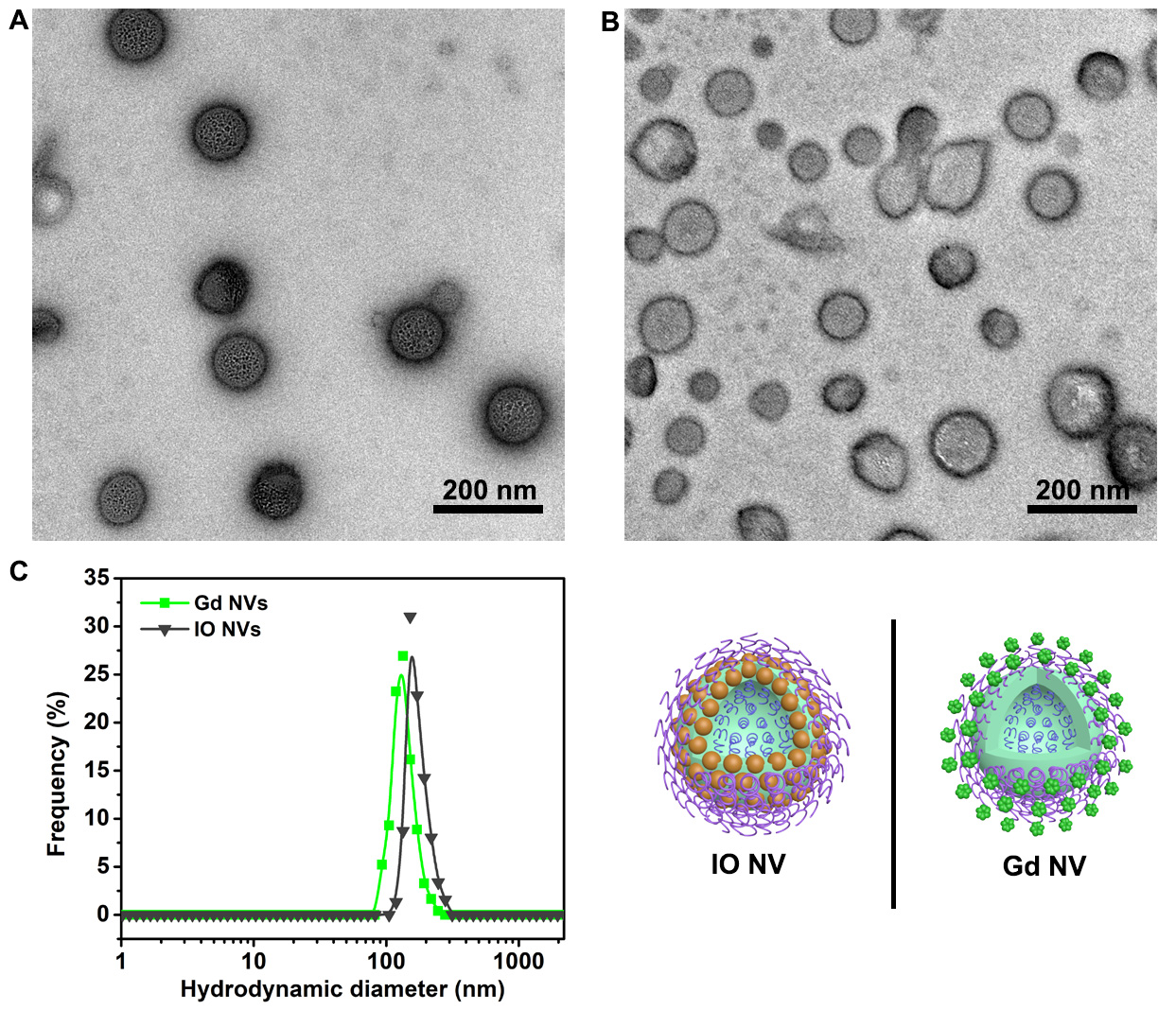


**Supplementary Fig. S6 | TEM and DLS measurements of the IO NVs and Gd NVs.** (A, B) TEM images of the IO NVs and Gd NVs, respectively. Scale bar 200 nm. (C) DLS measurements of the IO NVs (black) and Gd NVs (green) in water solution. The cartoon images (lower right) of IO NV and Gd NV are shown.


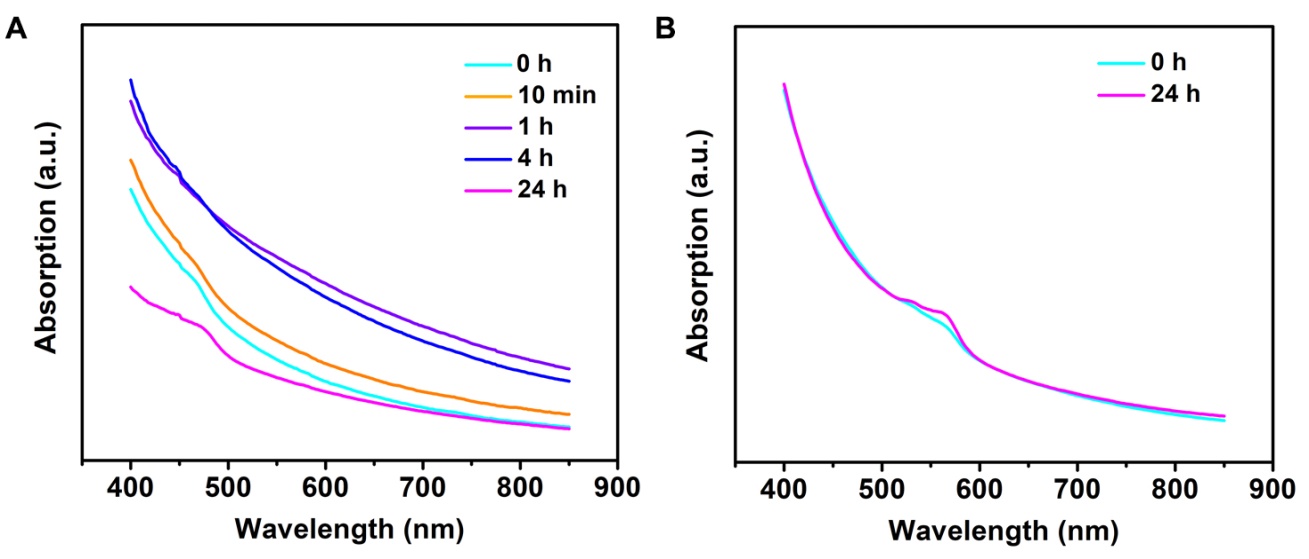


**Supplementary Fig. S7 | Time-dependent UV-vis absorption of the blank NVs.** (A) The blank NVs were incubated with H_2_O_2_ (10 mM) and the Uv-vis absorption was measured at different incubation time until 24 h. (B) The blank NVs were incubated with H_2_O_2_ (0.5 mM) the Uv-vis absorption was measured at different incubation time of 0 and 24 h.


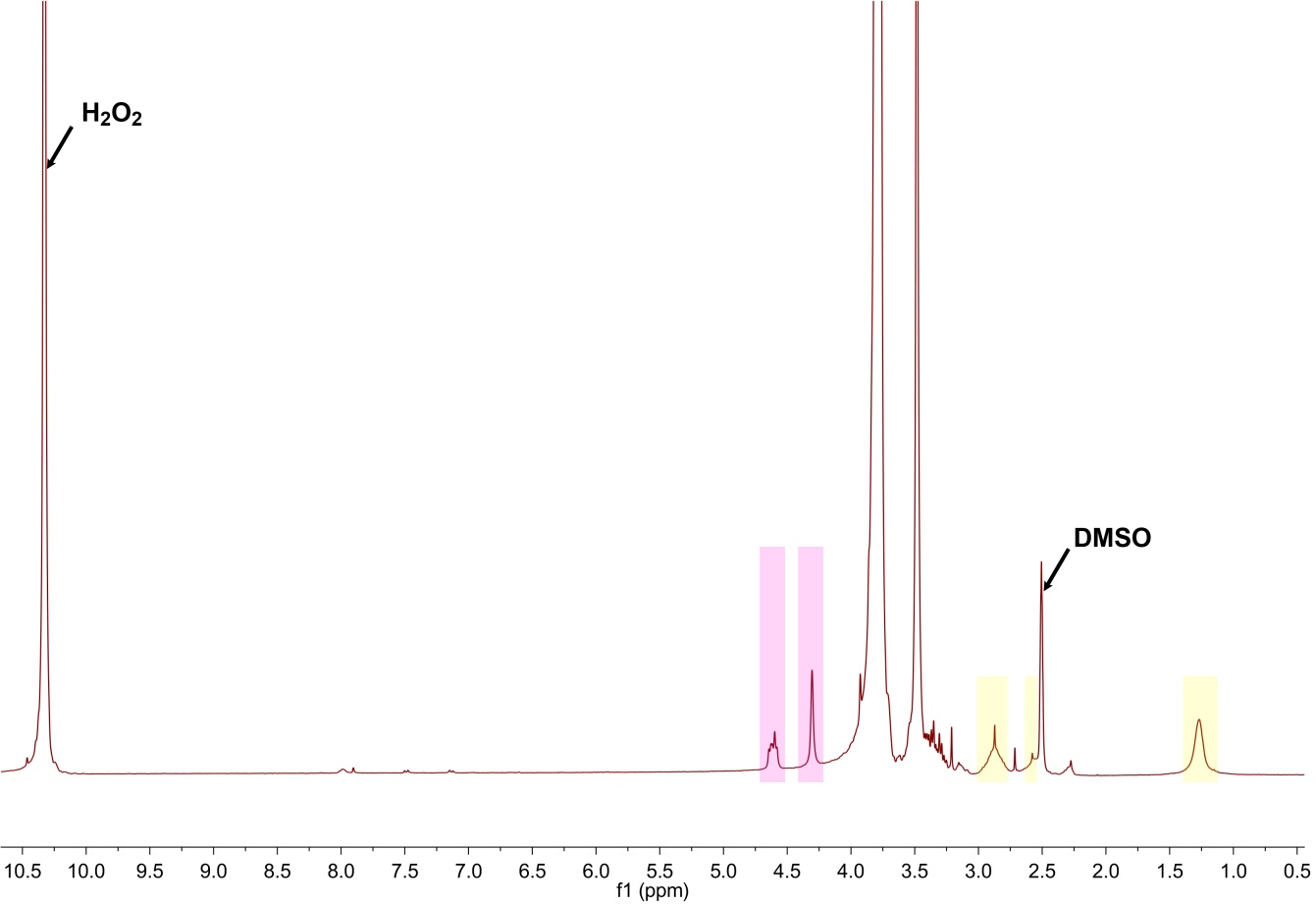


**Supplementary Fig. S8 | The proton NMR spectrum of the triblock polymers after oxidation.** The PEG_750_-PPS-PEG_1000_-NH_2_ triblock copolymers were treated with H_2_O_2_ (10 mM) for 24 h before the proton NMR measurement. The shrinkage of original chemical shifts for the PPS backbone (yellow squares) and the appearance of new chemical shifts (pink squares) indicate the oxidation of the thioethers in the PPS backbone.


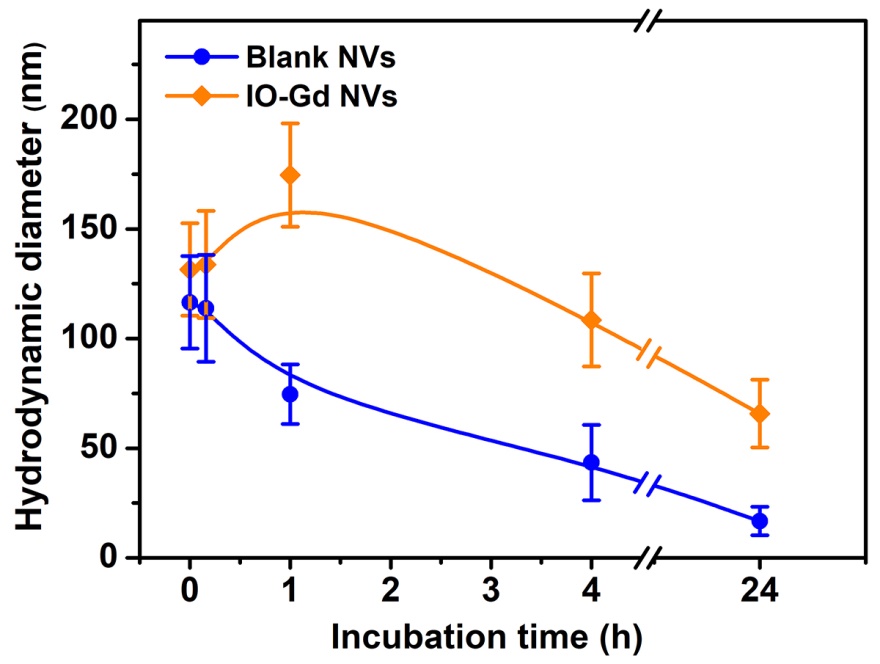


**Supplementary Fig. S9 | DLS measurements of the blank NVs and IO-Gd NVs.** The blank NVs and IO-Gd NVs were incubated with NaCl, MPO (5 U/mL), and H_2_O_2_ (500 µM). The hydrodynamic diameters were measured at different incubation time until 24 h. The experiments were independently and triply repeated (n = 3, data represents mean ± s.d.).


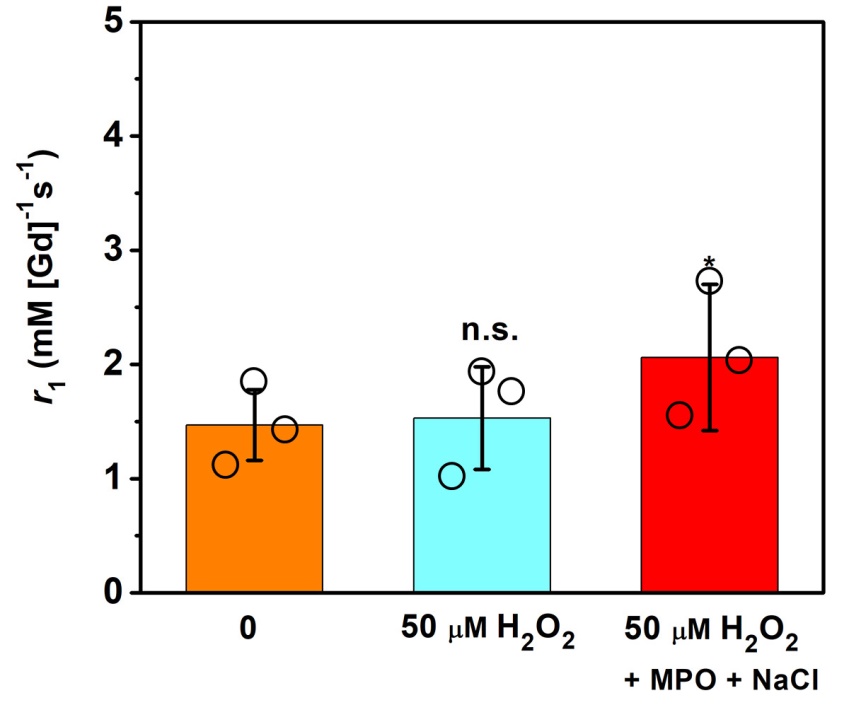


**Supplementary Fig. S10 | The *r*_1_ values of the IO-Gd NVs (Fe:Gd = 35.5:1) at 50 µM of H_2_O_2_.** The MPO (5 U/mL) and NaCl (500 mM) were used as implied. The experiments were independently and triply repeated (n = 3, data represents mean ± s.d., *p = 0.044; one-tailed paired t-tests). “n.s.” indicates no significance.


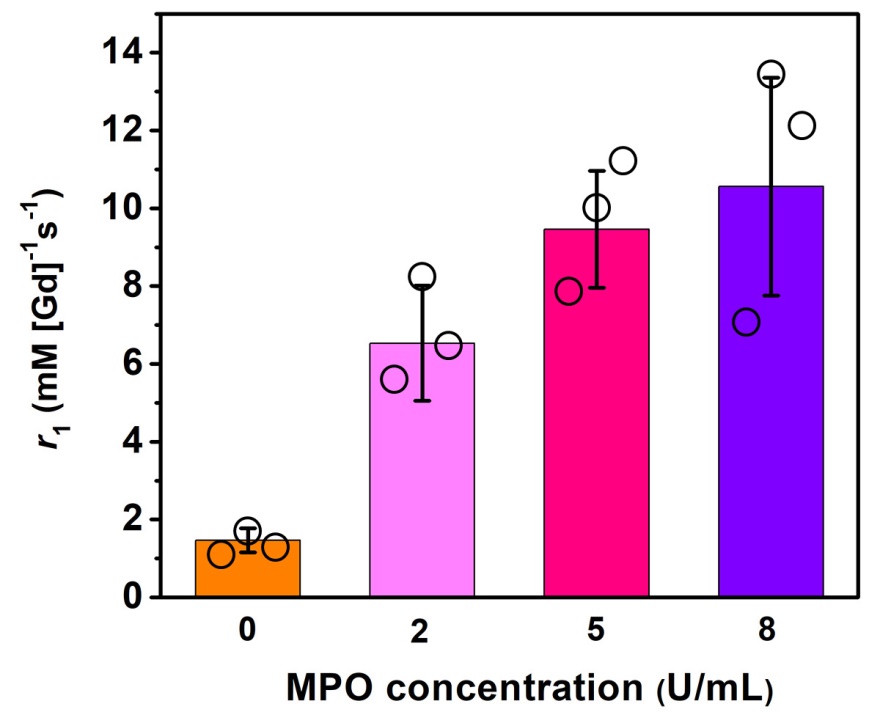


**Supplementary Fig. S11 | The *r*_1_ values of the IO-Gd NVs at different MPO concentrations.** The IO-Gd NVs (Fe:Gd = 35.5:1) were treated with MPO accompanied with 500 µM of H_2_O_2_ and NaCl (500 mM) under the same condition. The experiments were independently and repeated three times.­­ Data represents mean ± s.d.


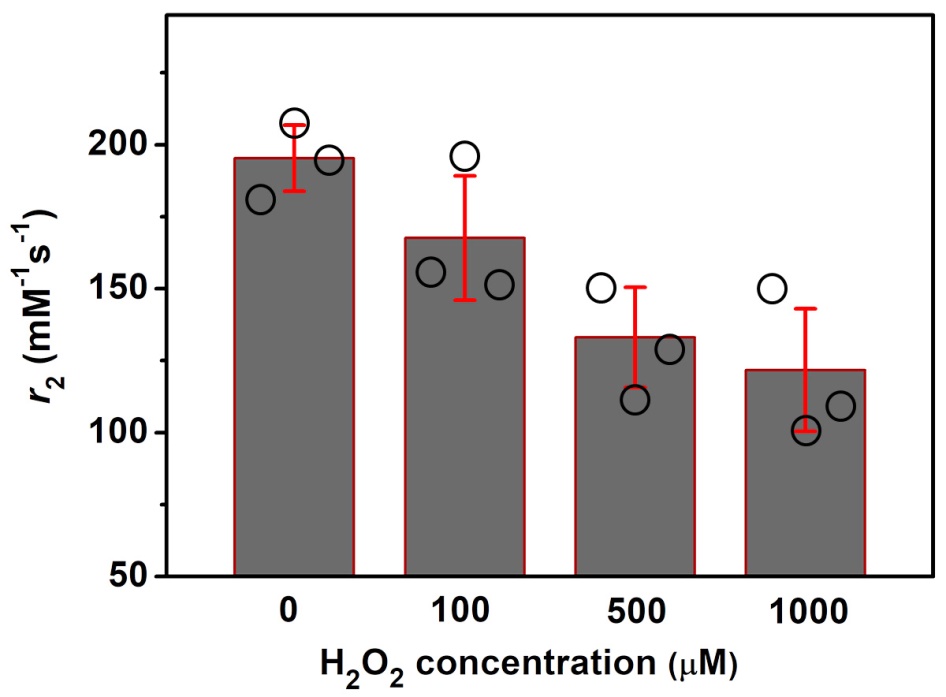


**Supplementary Fig. S12 | The *r*_2_ values of the IO-Gd NVs at different conditions.** The IO-Gd NVs (Fe:Gd = 35.5:1) were incubated with different concentrations of H_2_O_2_ (0, 100, 500, and 1000 µM) and with the same amount of NaCl and MPO (5 U/mL). The Fe concentrations were used for the *r*_2_ calculations. The experiments were repeated independently and triply (n = 3, data represents mean ± s.d.).


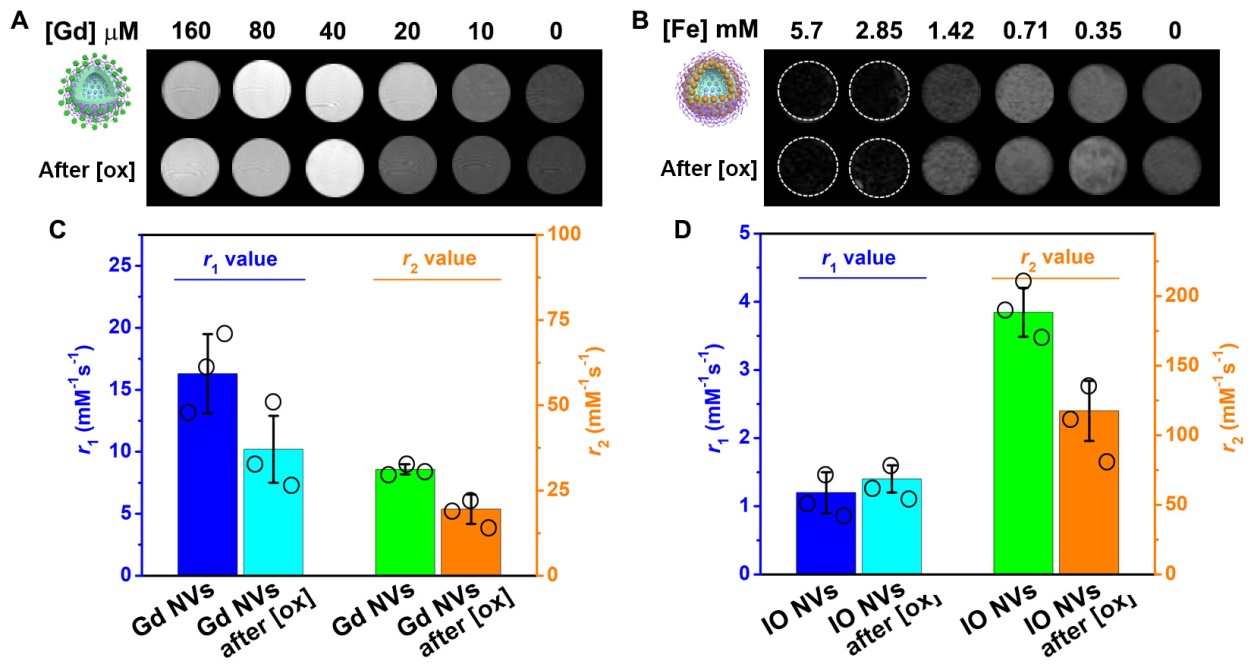


**Supplementary Fig. S13 | The *T*_1_ MRI study of the Gd NVs and IO NVs.** (A, C) The *T*_1_ phantom images and the *r*_1_ and *r*_1_ values of the Gd NVs before and after oxidation (ox) in the presence of H_2_O_2_ (1000 µM), NaCl and MPO (5 U/mL). (B, D) The *T*_1_ phantom images and the *r*_1_ and *r*_1_ values of the Fe NVs before and after oxidation (ox) in the presence of H_2_O_2_ (1000 µM), NaCl and MPO (5 U/mL). The Gd and Fe concentrations were used for calculating the *r*_1_ and *r*_2_ values, respectively. The experiments were repeated independently and triply (n = 3, data represents mean ± s.d.).


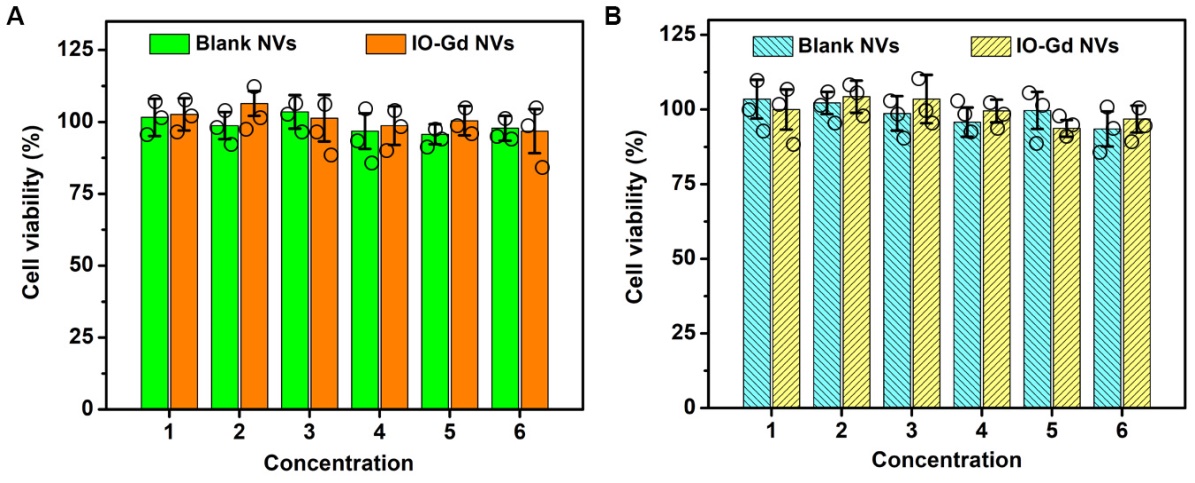


**Supplementary Fig. S14 | Biocompatibility study of the blank NVs and IO-Gd NVs.** (A, B) The blank NVs and IO-Gd NVs were incubated with U87MG and 4T1 cells, respectively, for 48 h before the viability measurements. The concentrations 1-6 represents to the concentration of blank NVs or IO-Gd NVs from 25, 50, 100, 200, 400 and 800 µg/mL. Data represents mean ± s.d.


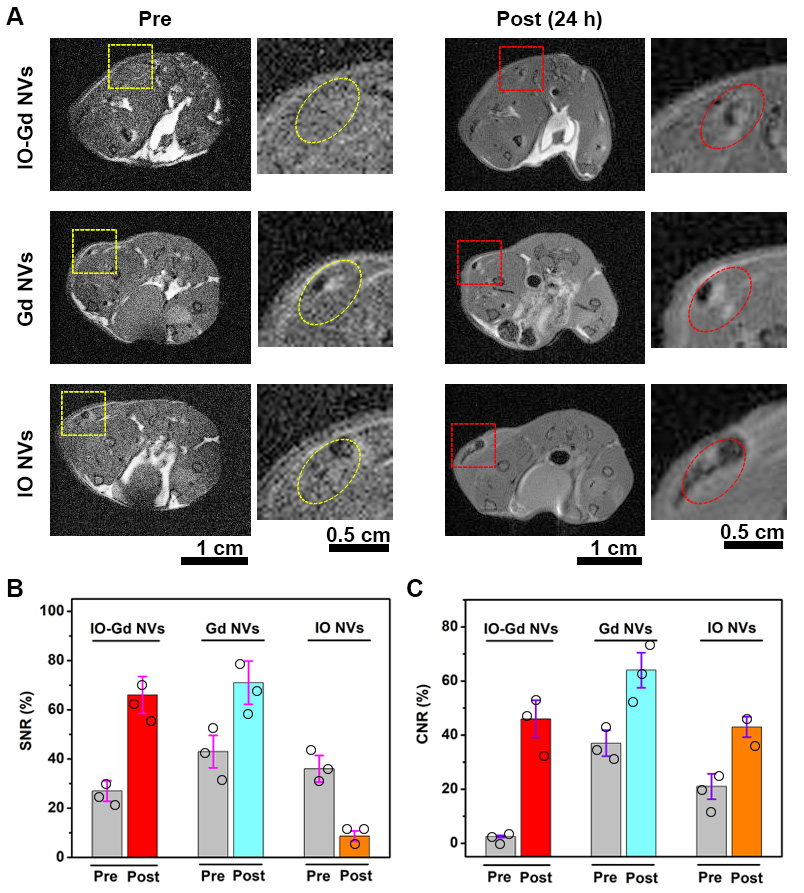


**Supplementary Fig. S15 | The aiMRI of inflammation in mouse muscle model.** (A) MR images of pre- and post-injection (p.i., 24 h) of IO-Gd NVs, Gd NVs or IO NVs in mouse muscle inflammation induced by turpentine injected 2 days before the MRI study. Contrast agents were intravenously injected with a dose of 5 µmol Gd per kg mouse body weight. The inflammation area was marked with squares and circles (right, magnified images). (B, C) The SNR and CNR of the inflammation foci at pre- and post-injection of different contrast agents, respectively. Data represents mean ± s.d. (n = 3 biologically independent animals).


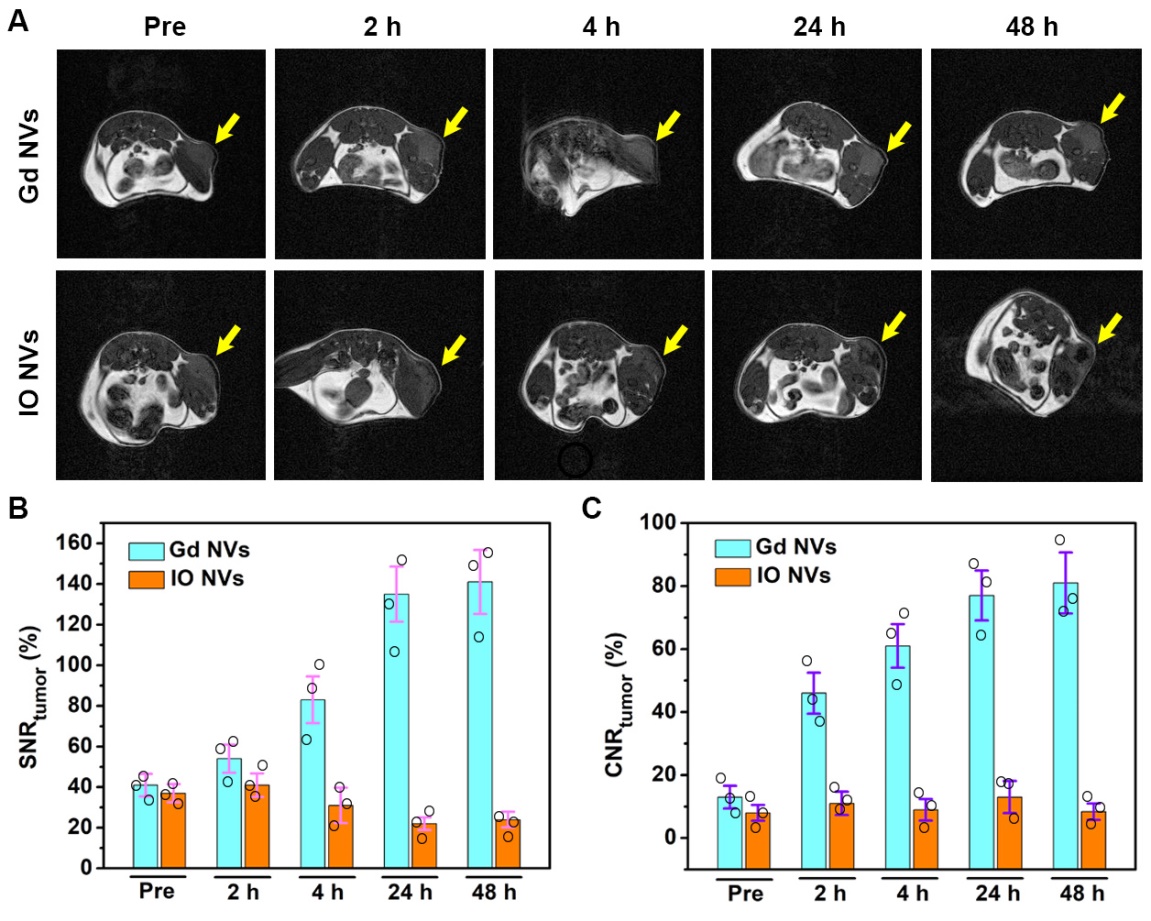


**Supplementary Fig. S16 | The aiMRI study of mouse tumor after X-ray irradiation.** (A) MR images of mouse tumor after X-ray irradiation (8 Gy) which was performed 24 h before the MRI acquisition. The IO NVs or Gd NVs were used as contrast agents and the post-injection (p.i., 2, 4, 24, 48 h) time points MRI was acquired subsequently. Tumor is indicated by the yellow arrows. (B, C) Semi-quantitative analysis of the SNRtumor and CNRtumor from MRI images. Data represents mean ± s.d. (n = 3 biologically independent animals).

**
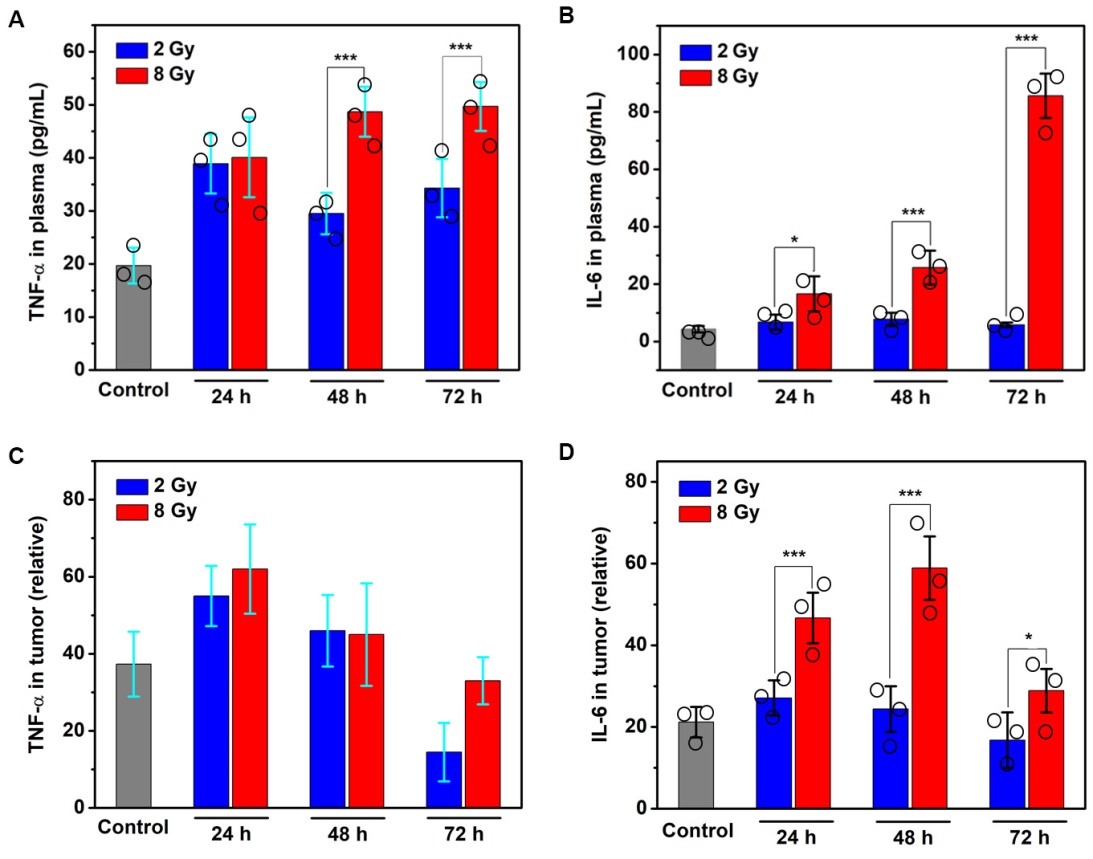
**

**Supplementary Fig. S17 |** **ELISA analysis of TNF-α (A, C) and IL-6 (B, D) in mouse plasma and tumor.** Mice were treated with 0 (control), 2, or 8 Gy X-ray irradiation and sacrificed for analysis of IL-6 and TNF-α in the tumor and in the plasma by ELISA kits. The data was obtained based on three independent experiments for each group (n = 3/group). *P = 0.041 and 0.017 for Fig. B and D, respectively; ***P < 0.001; one-tailed paired t-tests. Data represents mean ± s.d.


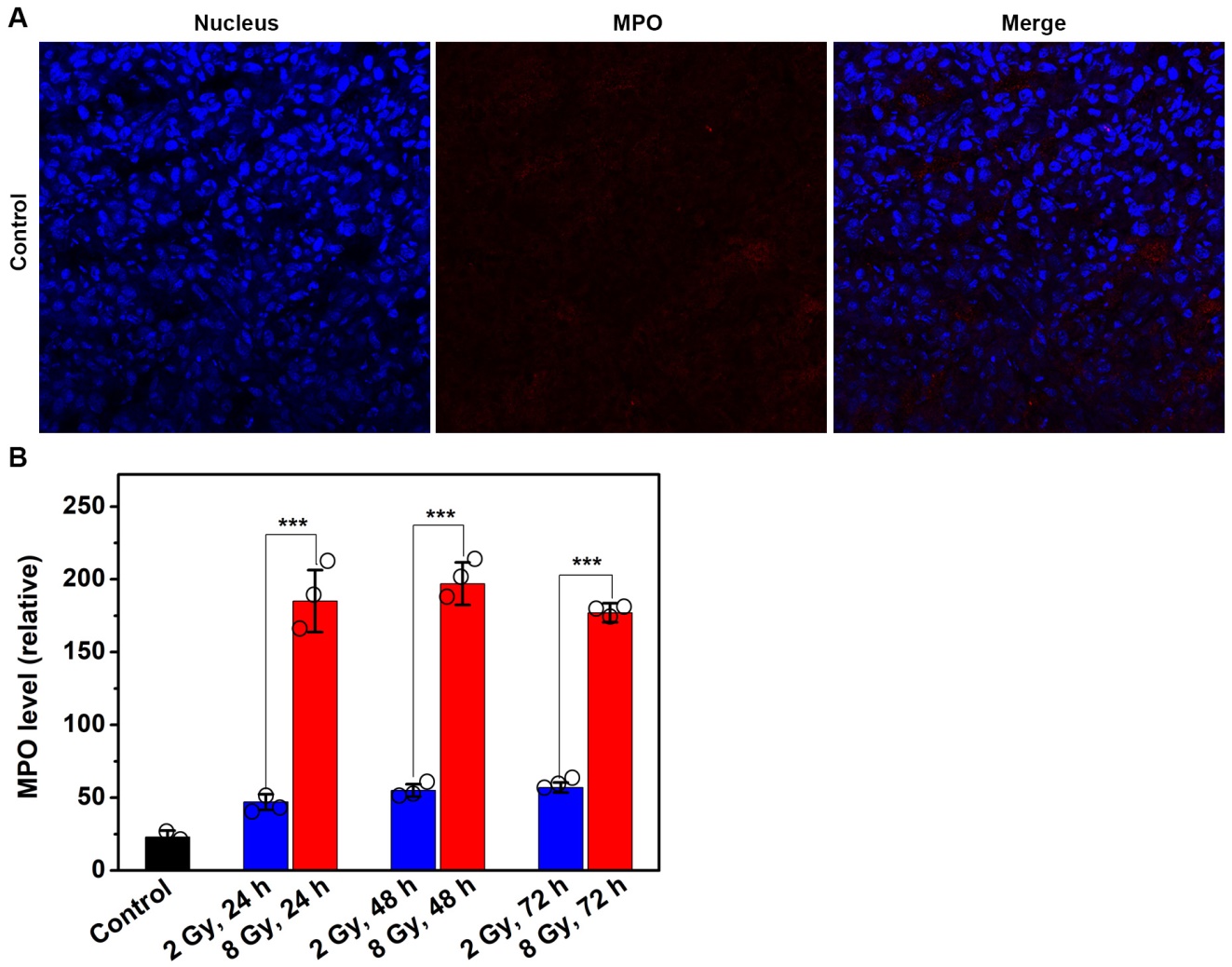


**Supplementary Fig. S18 | Immunofluorescence staining of MPO level in mouse tumor.** (A) The MPO and nucleus staining of tumor slice from untreated control group. Scale bar 100 µm. (B) The semi-quantitative analysis of the MPO levels for different groups. This result is supplementary to the Fig. 4c in the main text. ***P < 0.001 (n = 3 biologically independent tissue samples per group); two-tailed homoscedastic t-tests. Data represents mean ± s.d.


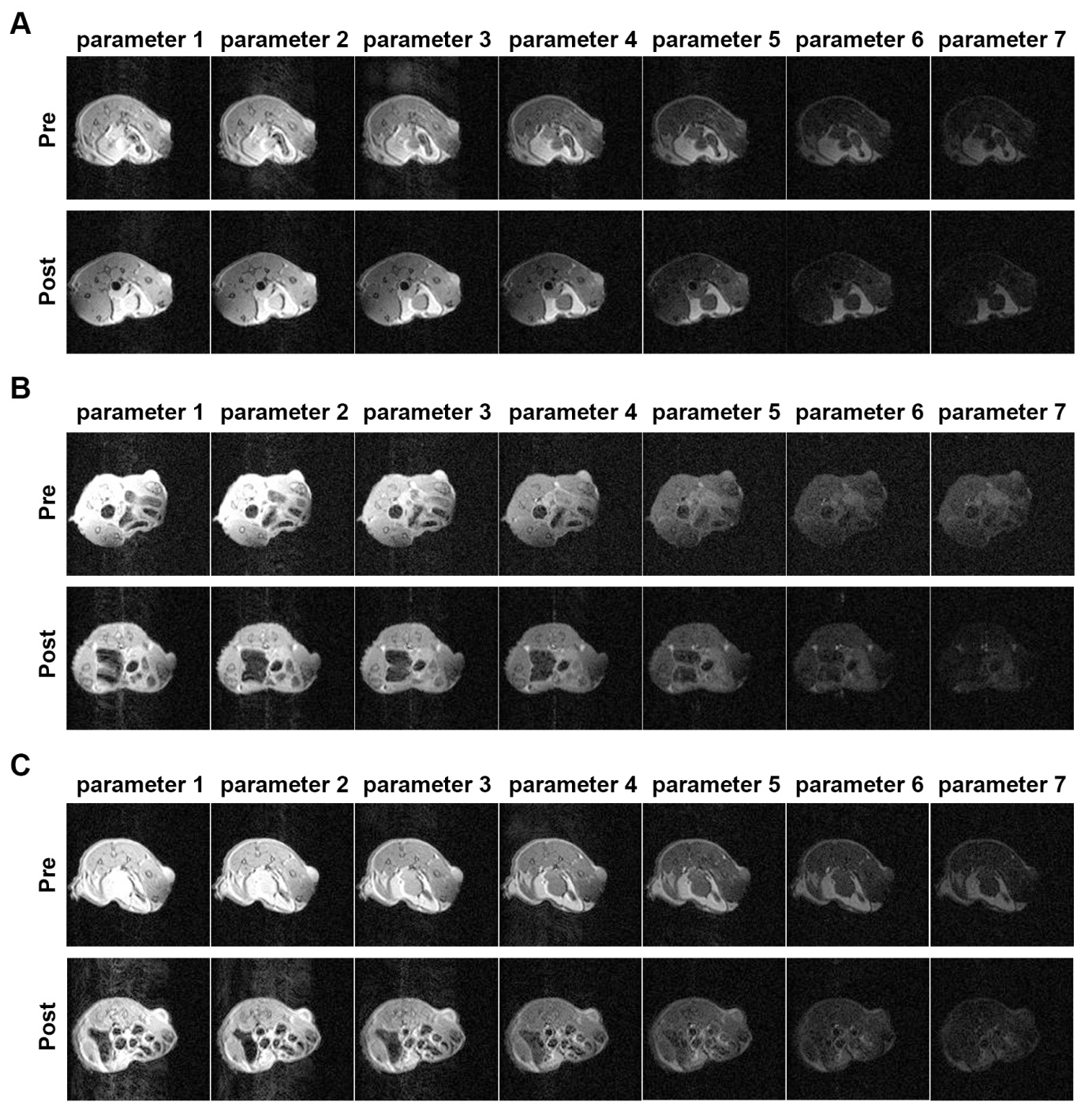


**Supplementary Fig. S19 |** **The** **multi-parametric *T*_1_ MR images.** (A-C) multi-parametric *T*_1_ MR images of mouse receiving 0 (A), 2 (B) or 8 (C) Gy irradiation. The IO-Gd NVs (Fe:Gd 35.5:1) were used as contrast agents. Parameters 1-7 represents to multiple TR = 6000, 4000, 2000, 1000, 500, 250, and 169.3 ms, respectively, and TE = 10.3 ms for all images. These results are supplementary to the Fig. 4d in the main text.


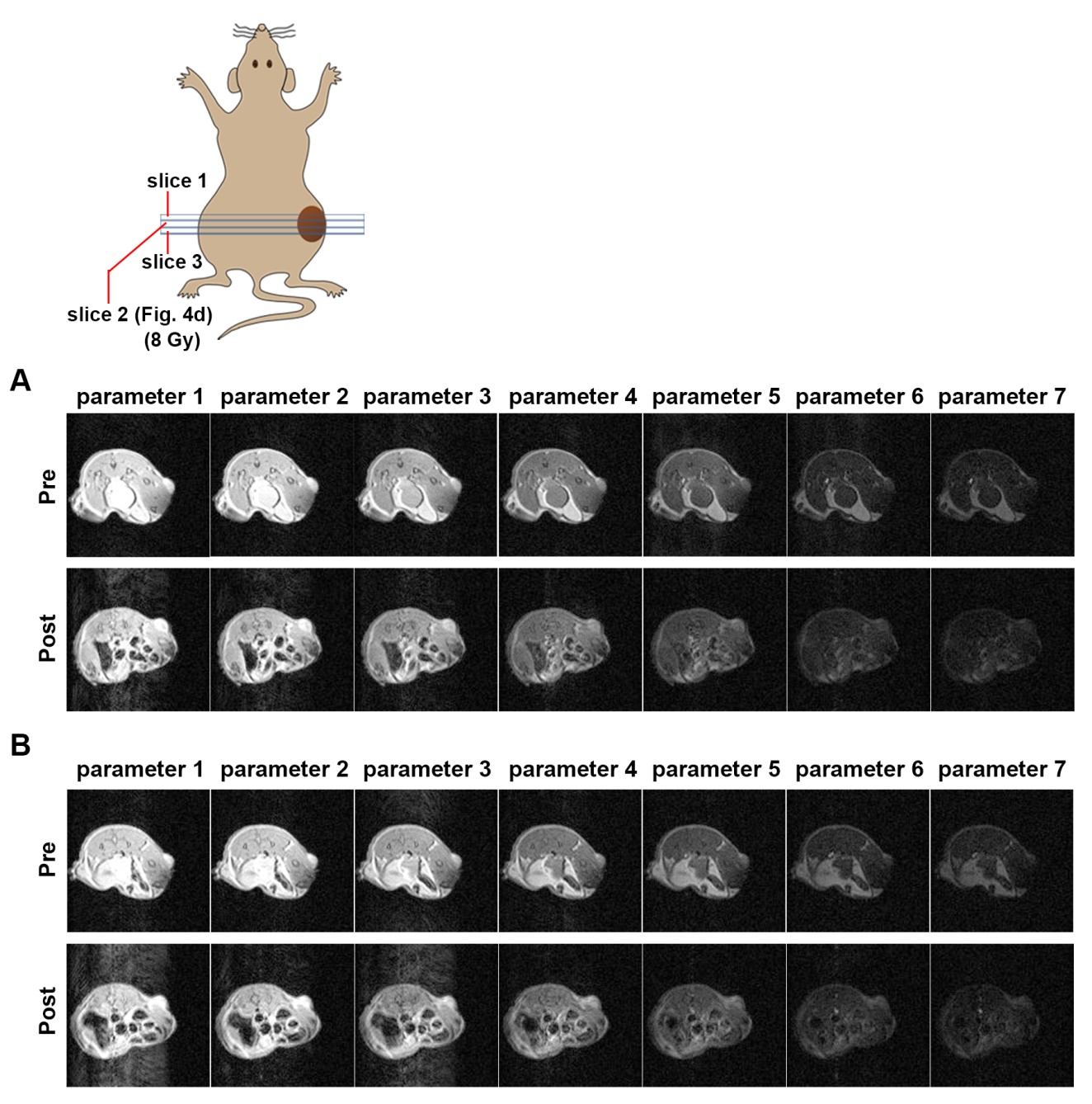


**Supplementary Fig. S20 | Additional multi-parametric *T*_1_ MR images.** (A, B) Additional multi-parametric *T*_1_ MR images of the adjacent tumor slices (slice 1 and 3) to the results shown in the Fig. 4d (8 Gy, slice 2). Parameter 1-7 represents to multiple TR = 6000, 4000, 2000, 1000, 500, 250, and 169.3 ms, respectively, and TE = 10.3 ms for all images.


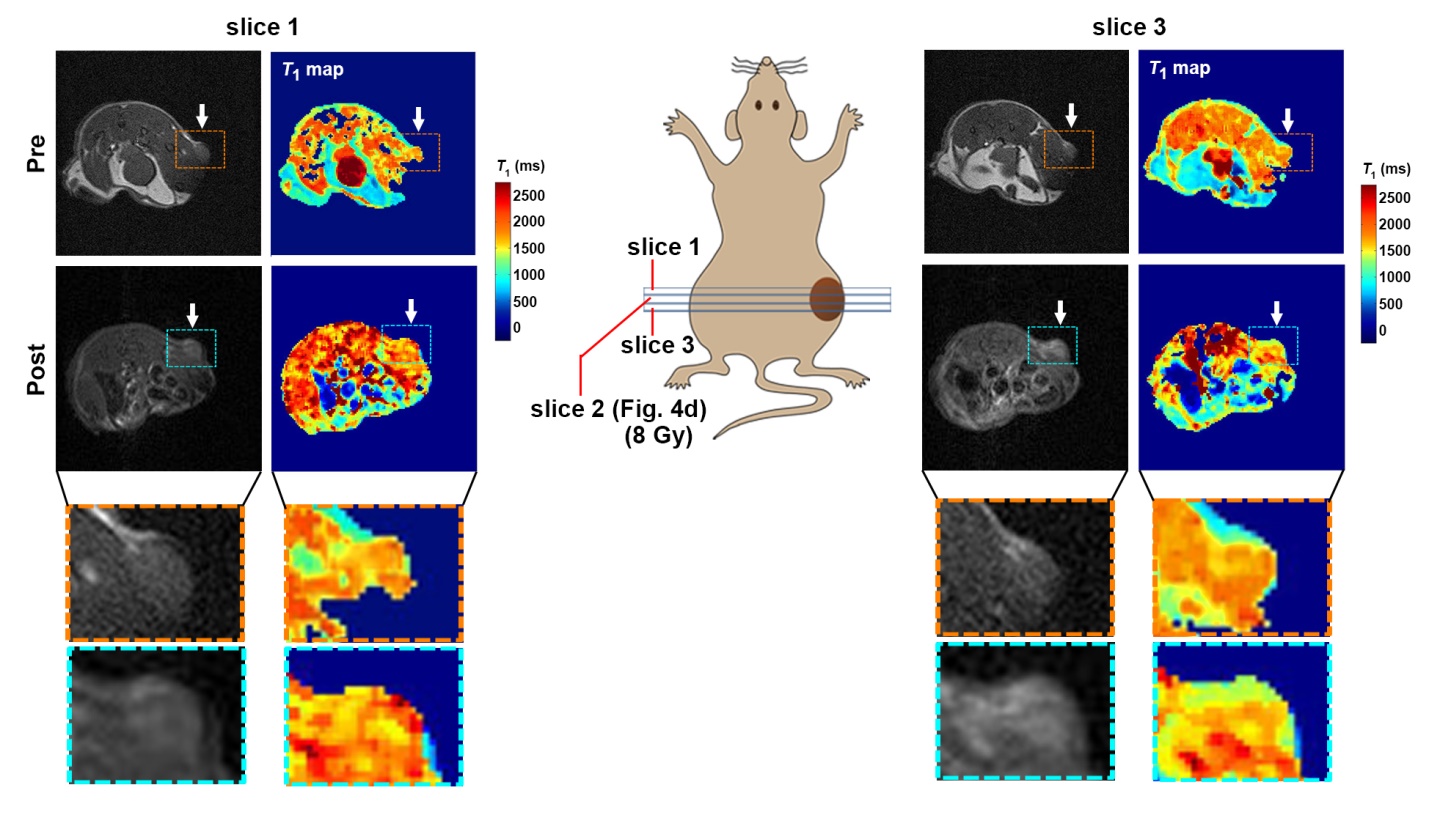


**Supplementary Fig. S21 |** **Additional *T*_1_ MRI relaxation time maps.** The *T*_1_ MRI maps were reconstructed from the multi-parametric *T*_1_ MR images shown in the Supplementary Fig. S17. The magnified *T*_1_-weighted MR images and the *T*_1_ relaxation maps indicate the heterogeneous relaxation changes in the tumor.


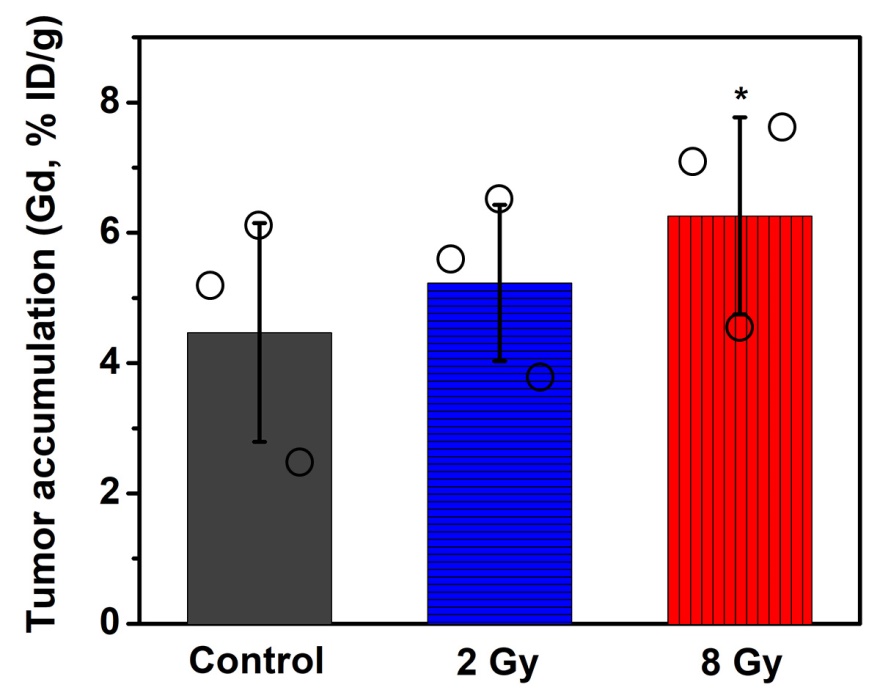


**Supplementary Fig. S22 | Tumor accumulation measurements of the IO-Gd NVs.** The IO-Gd NVs (Fe:Gd 35.5:1) were intravenously injected into mouse tail vein with a dose of 10 µmol [Gd]/kg mouse body weight. Mouse tumors were collected at 24 h p.i. of the IO-Gd NVs which was 48 h after receiving 2 or 8 Gy irradiation. *P = 0.027 (n = 3, data represents mean ± s.d., one-tailed paired t-tests).


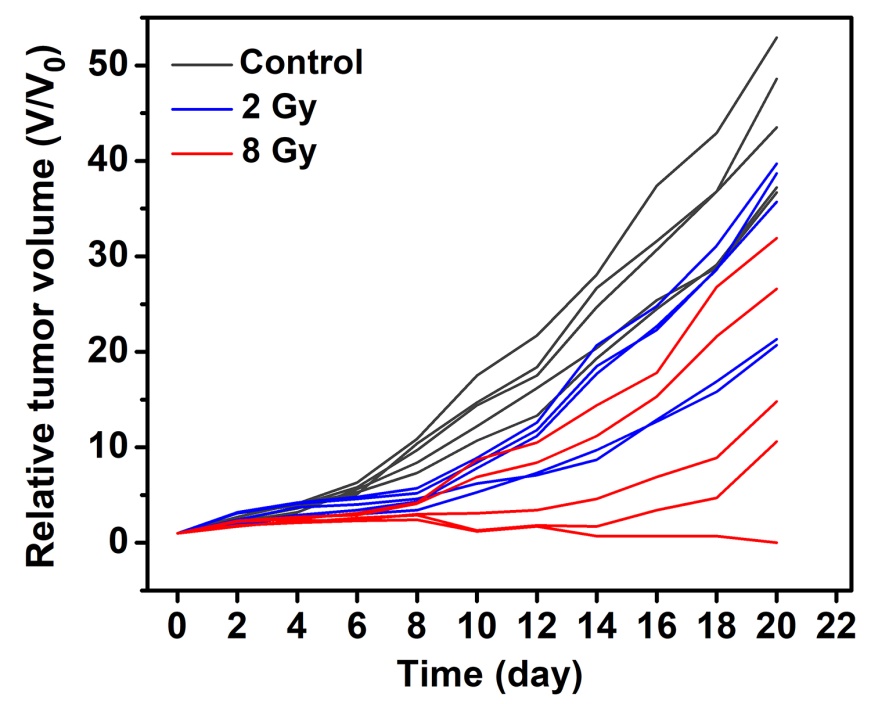


**Supplementary Fig. S23 | Individual mouse tumor growth curves.** The U87 MG mouse tumor groups were treated with control, 2 Gy, or 8 Gy irradiation at the day 0. This data is supplementary to the Fig. 4f in the main text.


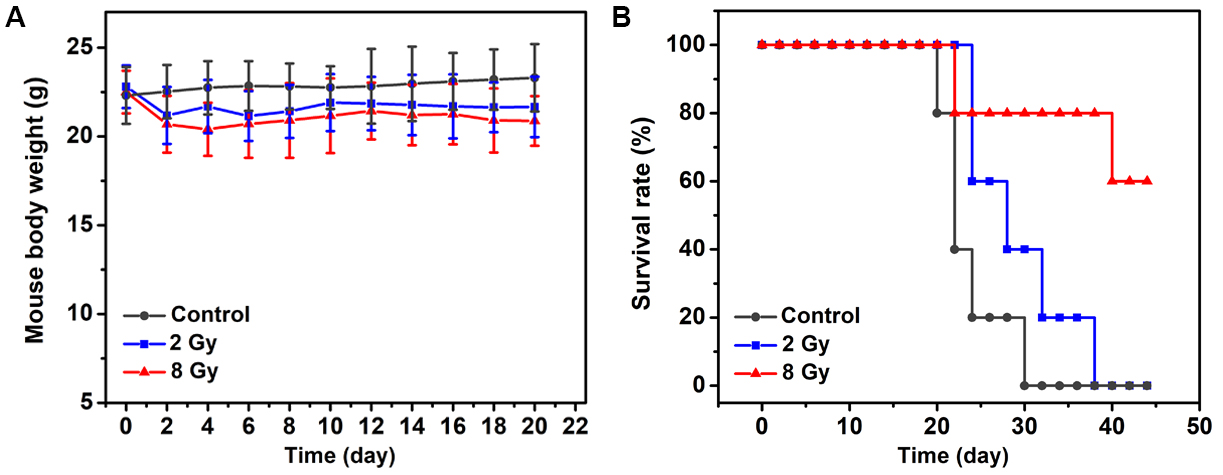


**Supplementary Fig. S24 | The mouse body weight and survival rate.** (A, B) The mouse body weight (A) and survival rate (B) of mouse U87 MG tumor groups receiving 0 (control), 2 or 8 Gy irradiation at day 0, respectively (n = 5 for each group, data represents mean ± s.d.).


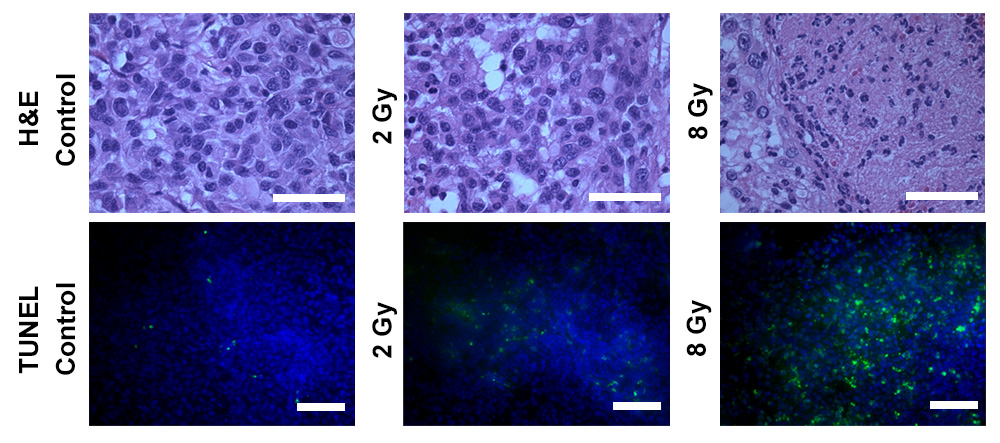


**Supplementary Fig. S25 | Representative H&E and TUNEL staining results.** The U87 MG tumor slices were dissected from mice after different doses of RT. Scale bar: 100 µM for all images.


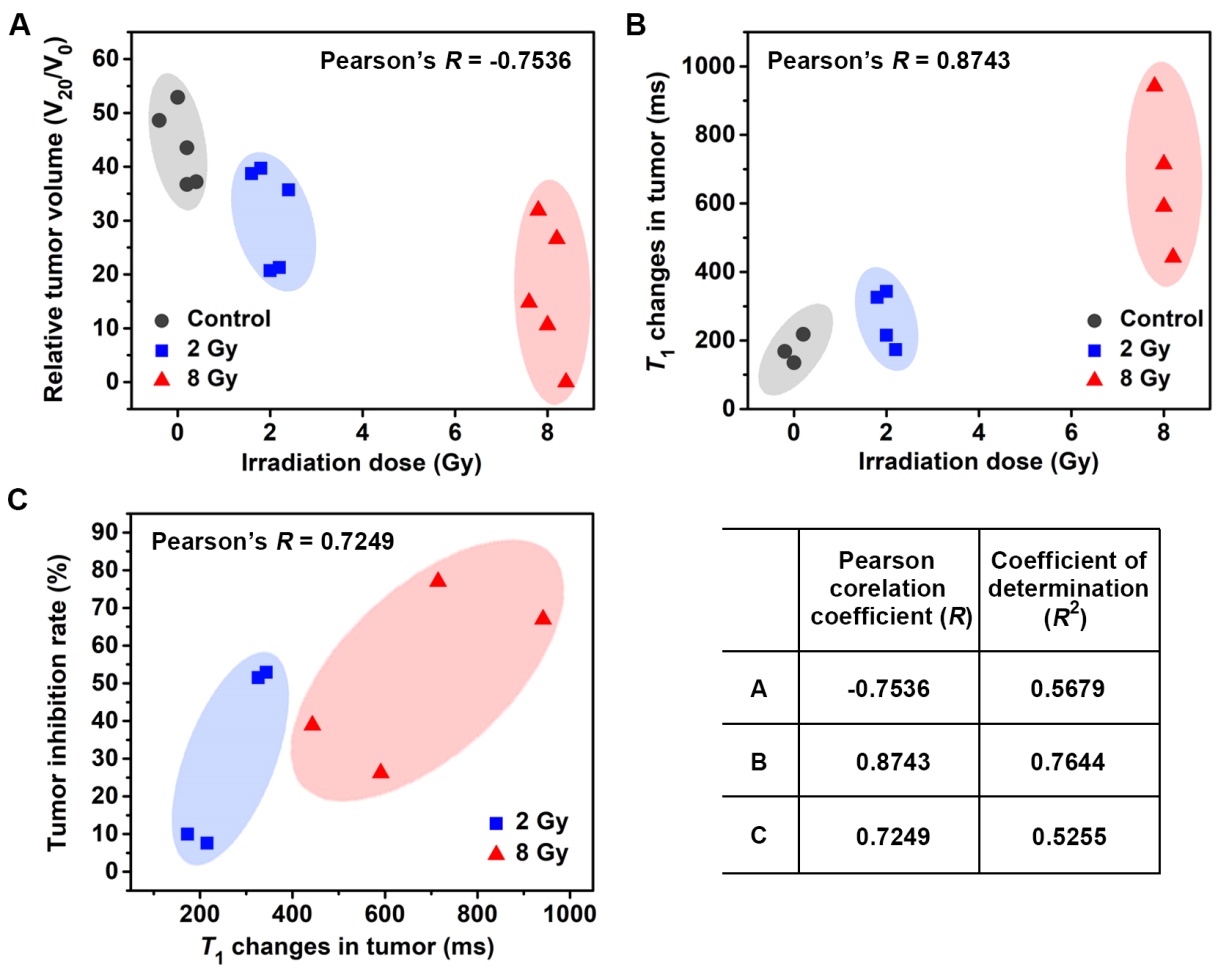


**Supplementary Fig. S26 | The Pearson’s correlation analysis of RT in U87 MG tumor model.** (A-C) The correlations between the irradiation doses, *T*_1_ relaxation time changes in tumors, and tumor inhibition rate were plotted. The Pearson’s correlation coefficient (*R*) and the coefficient determination (*R*^2^) are summarized (lower right). These results indicate strong correlations between the inflammatory responses and the anti-tumor effect in RT, which can be quantified by the *T*_1_ relaxation time changes in the aiMRI.


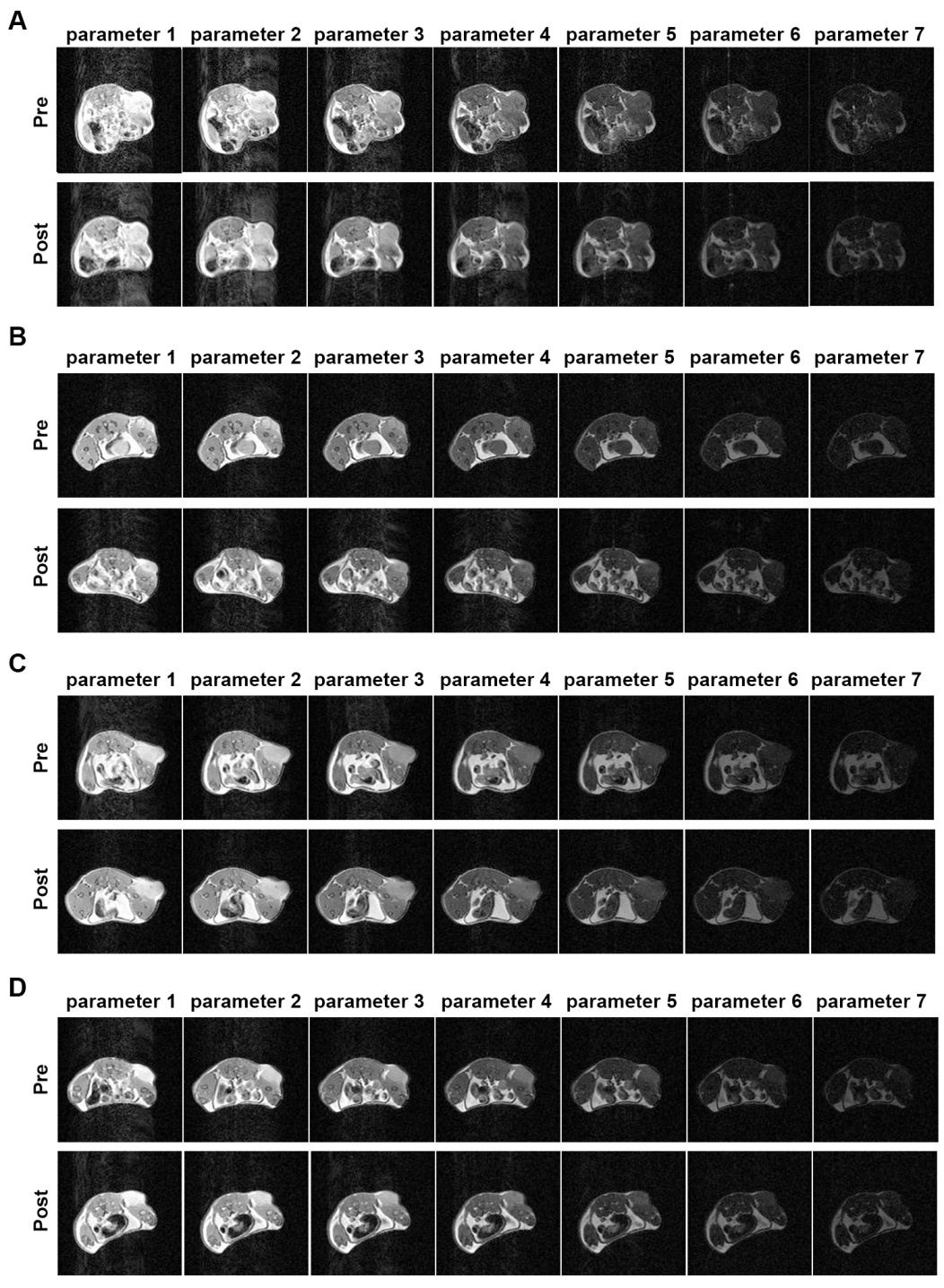


**Supplementary Fig. S27 | The multi-parametric *T*_1_ MR images of 4T1 tumors.** (A-D) Multi-parametric *T*_1_ MR images of mouse receiving 0 (A), RT only (B), RT + aLy6G (C), or RT + DPI (D). Parameters 1-7 represents to TR = 6000, 4000, 2000, 1000, 500, 250, and 144.195 ms, respectively, and TE = 11.5 ms for all images. These results are supplementary to the Fig. 5b-d in the main text.


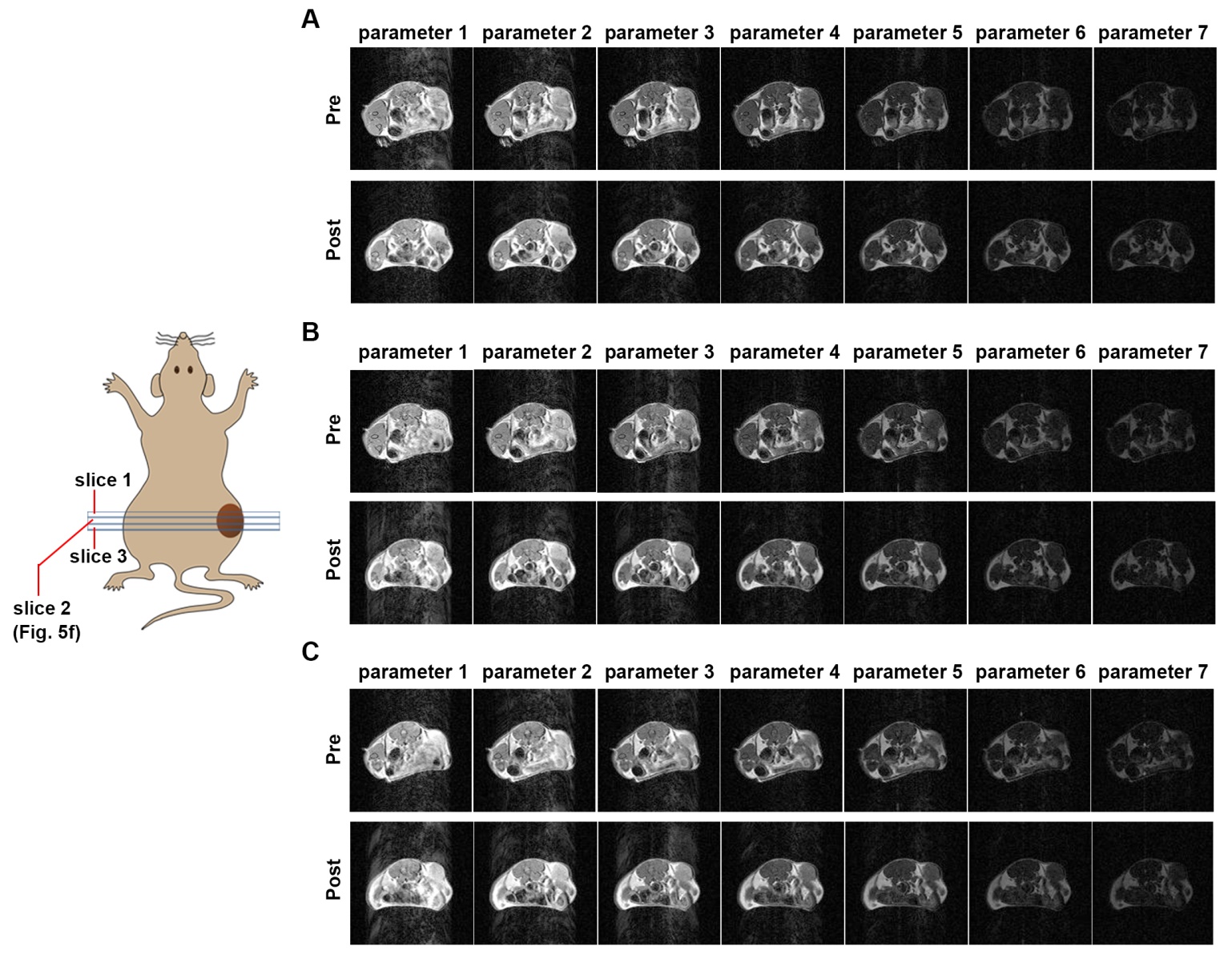


**Supplementary Fig. S28 | The multi-parametric multi-slice *T*_1_ MR images.** (A-C) Multi-parametric and multi-slice *T*_1_ MR images of mouse 4T1 tumor receiving RT + G-CSF treatment. Parameters 1-7 represents to TR = 6000, 4000, 2000, 1000, 500, 250, and 144.195 ms, respectively, and TE = 11.5 ms for all images. The MR images of slice 2 are supplementary to the Fig. 5f in the main text.


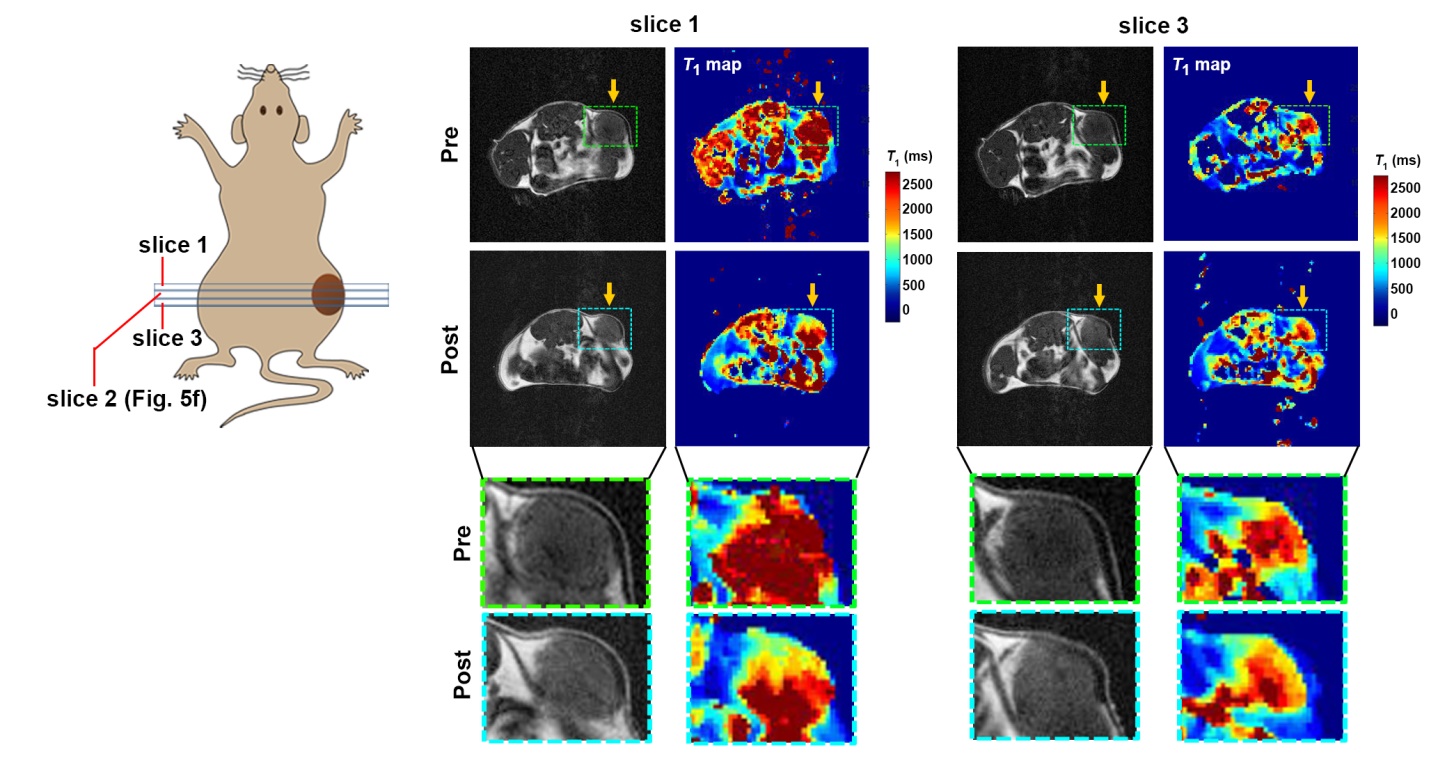


**Supplementary Fig. S29 | Additional multi-slice *T*_1_ MRI relaxation time maps.** The *T*_1_ MRI maps were reconstructed from the multi-parametric *T*_1_ MR images shown in the Supplementary Fig. S25. The mice bearing 4T1 tumor were treated with RT + G-CSF and were used for aiMRI at pre- and post-injection of contrast agents.


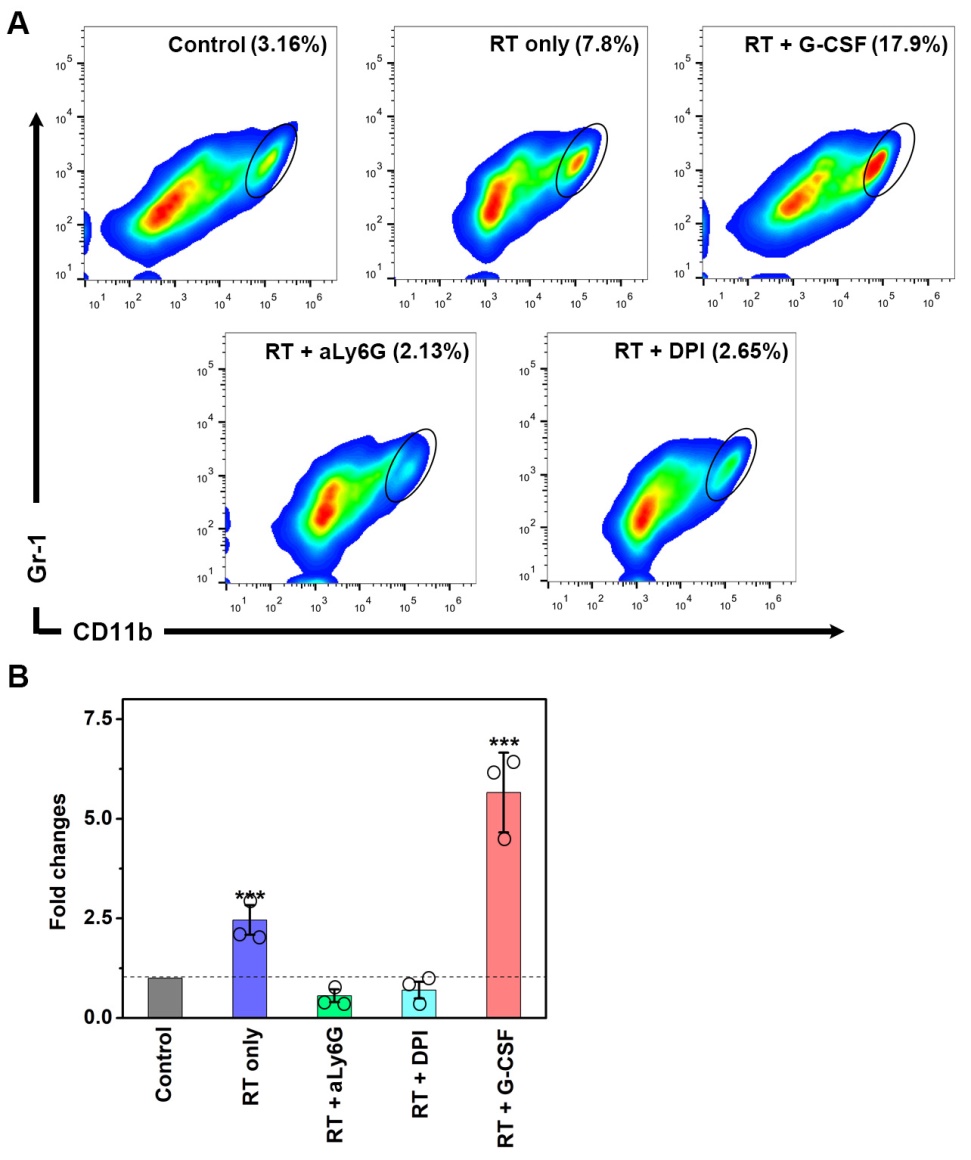


**Supplementary Fig. S30 | Flow cytometry analysis of CD11b^+^Gr-1^+^ neutrophils in tumor.** The Balb/c mouse 4T1 tumors were dissected at day 5 after different treatments including control, RT only, RT + aLy6G, RT + DPI, and RT + G-CSF. (A) Isolated tumor cells were stained with Cy5-CD11b and APC-Gr-1 antibodies. (B) Analysis of the fold changes of the CD11b^+^Gr-1^+^ neutrophils for different groups with respect to the control (dash line). The results were based on independent triplicate experiments (n = 3, ***P < 0.001; data represents mean ± s.d., one-tailed homoscedastic t-tests).


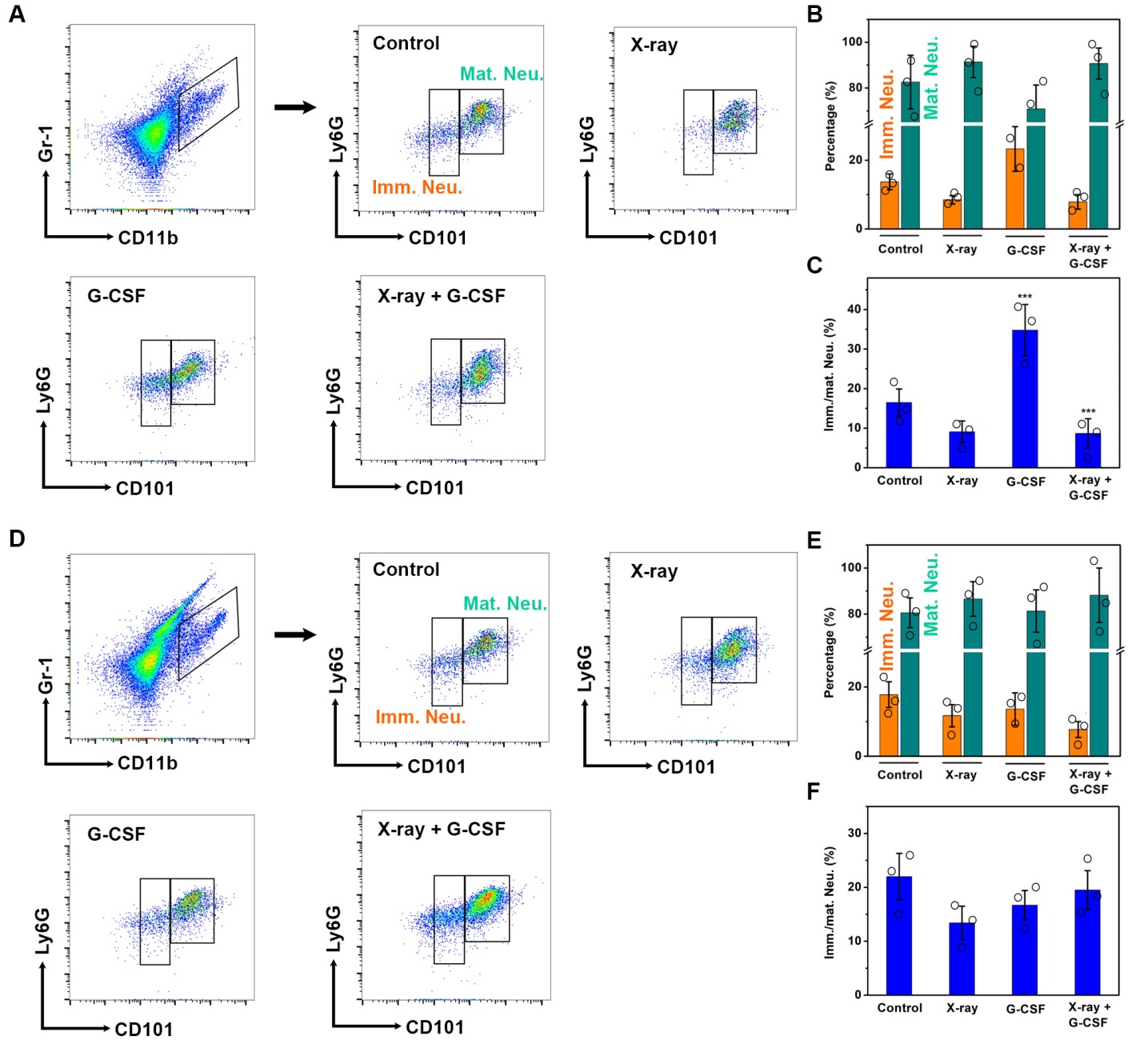


**Supplementary Fig. S31 | Flow cytometry analysis of immature and mature neutrophils after different treatments.** (A-C) The flow cytometry profiles and the analysis of the immature and mature neutrophils in the tumor after different treatments (control, X-ray, G-CSF, and X-ray + G-CSF). (D-F) The flow cytometry profiles and the analysis of the immature and mature neutrophils in blood after different treatments on tumors. The statistical analyses were based on triplicate experiments (n = 3/group, ***P < 0.001; data represents mean ± s.d., one-tailed paired t-test).


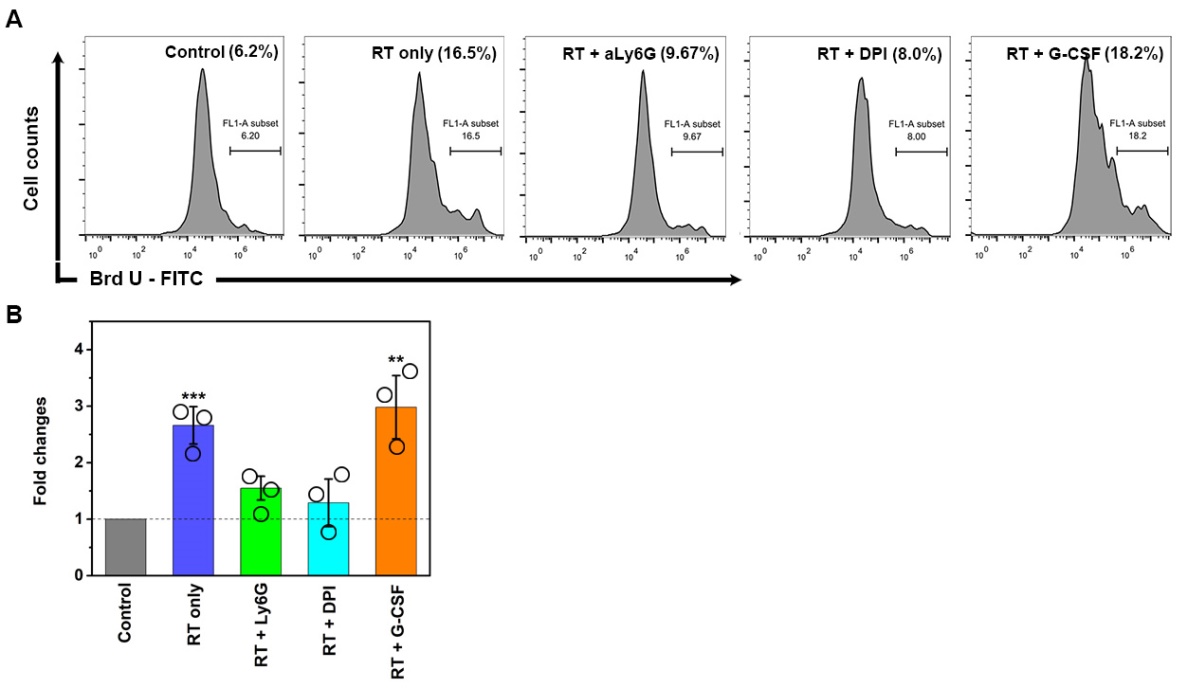


**Supplementary Fig. S32 | Analysis of apoptotic tumor cell death.** (A) Flow cytometry analysis of apoptotic death of tumor cells at day 5 after different treatments. (B) Quantitative analysis of the fold changes of apoptotic cells at day 5 after different treatments. These results were based on independent triplicate experiments (n = 3/group, data represents mean ± s.d., **P =0.0038, ***P < 0.001; one-tailed homoscedastic t-test).


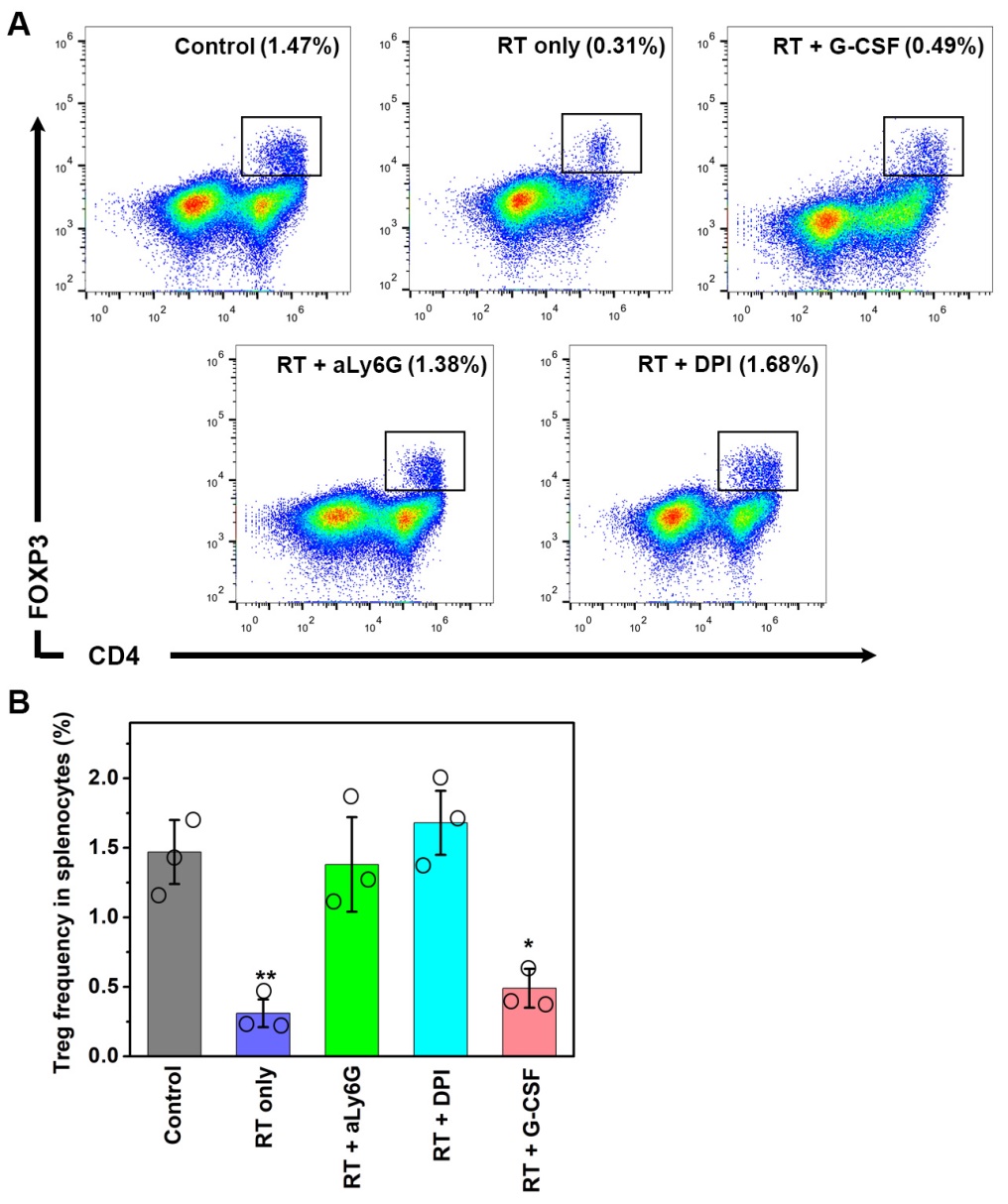


**Supplementary Fig. S33 | Flow cytometry analysis of Treg cells in splenocytes.** Balb/c mouse 4T1 tumors were dissected at day 5 after different treatments including control, RT only, RT + aLy6G, RT + DPI, and RT + G-CSF. (A) Isolated splenocytes from different treatment groups were stained using mouse Treg flow kit (FOXP3-Alexa Fluor 488/CD4-APC/CD25-PE). (B) Analysis of the FOXP3^+^CD4^+^ Treg cells in splenocytes. These results were based on independent triplicate experiments (n = 3/group). Data represents mean ± s.d. (**P = 0.0056, *P = 0.022, one-tailed paired t-test).


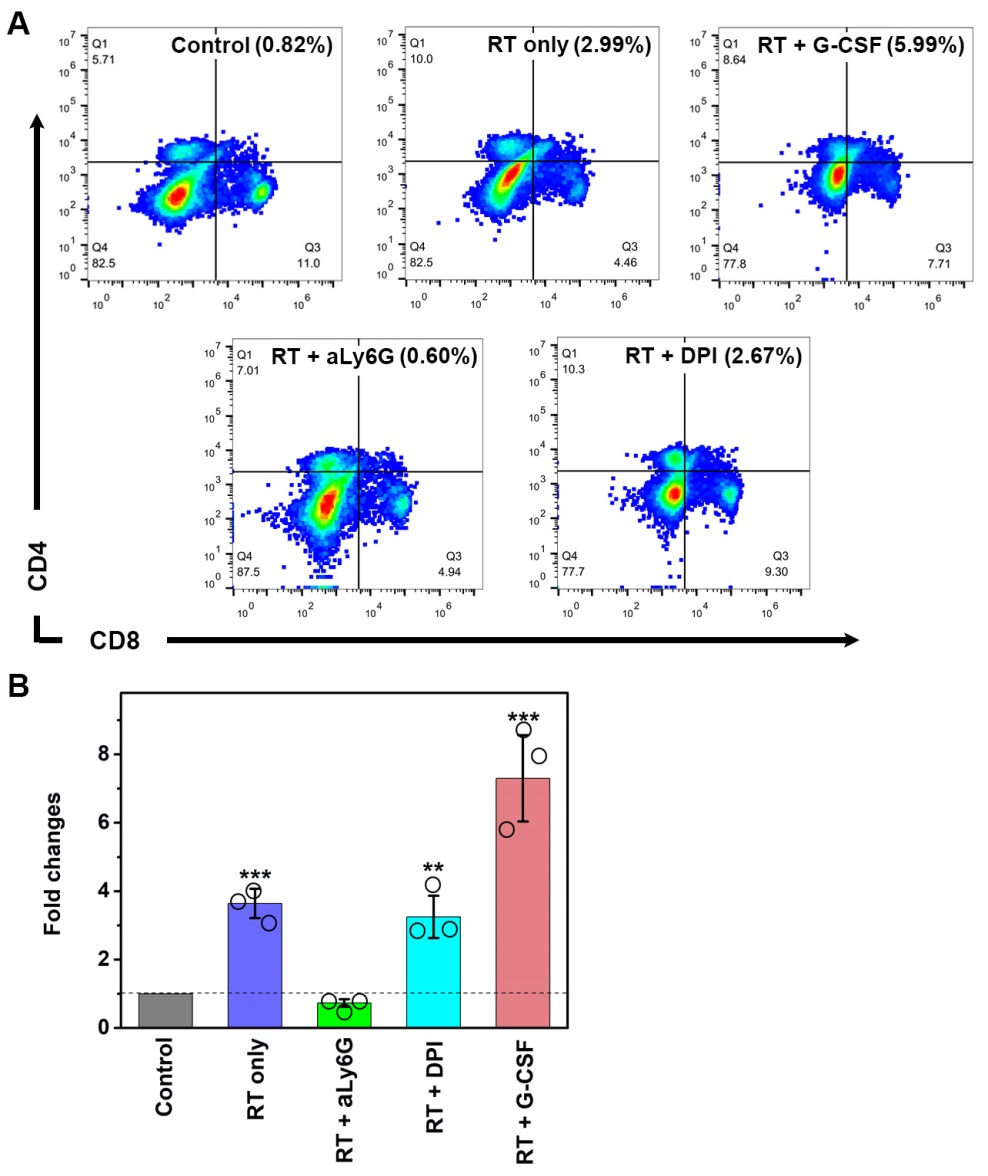


**Supplementary Fig. S34 | Flow cytometry analysis of CD4^+^CD8^+^ T lymphocytes.** Balb/c mouse 4T1 tumors were dissected at around day 18 after different treatments including control, RT only, RT + aLy6G, RT + DPI, and RT + G-CSF. (A) Isolated tumor cells were stained with PE-CD4 and APC-CD8 antibodies. (B) Analysis of the fold changes of CD4^+^CD8^+^ T lymphocytes for different groups with respect to the control (dash line). The results were based on independent triplicate experiments (n = 3/group, data represents mean ± s.d., **P = 0.0034, ***P < 0.001; one-tailed homoscedastic t-test).


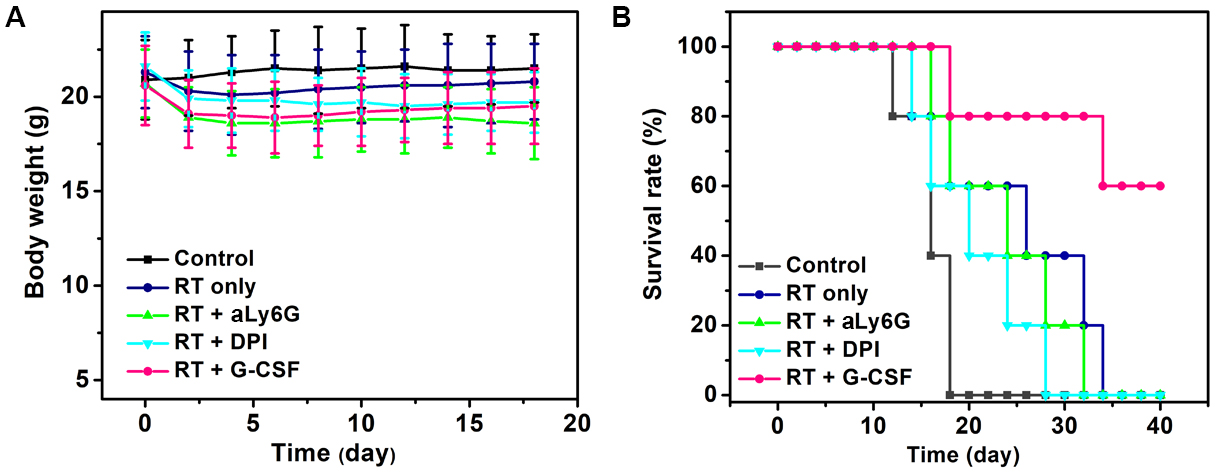


**Supplementary Fig. S35 | The RT study in Balb/c mouse 4T1 tumor models.** The mouse body weight (A) and survival rate (B) of Balb/c mouse 4T1 tumor models receiving control, RT only, RT + aLy6G, RT + DPI, or RT + G-CSF, respectively (n = 5/group, data represents mean ± s.d.).


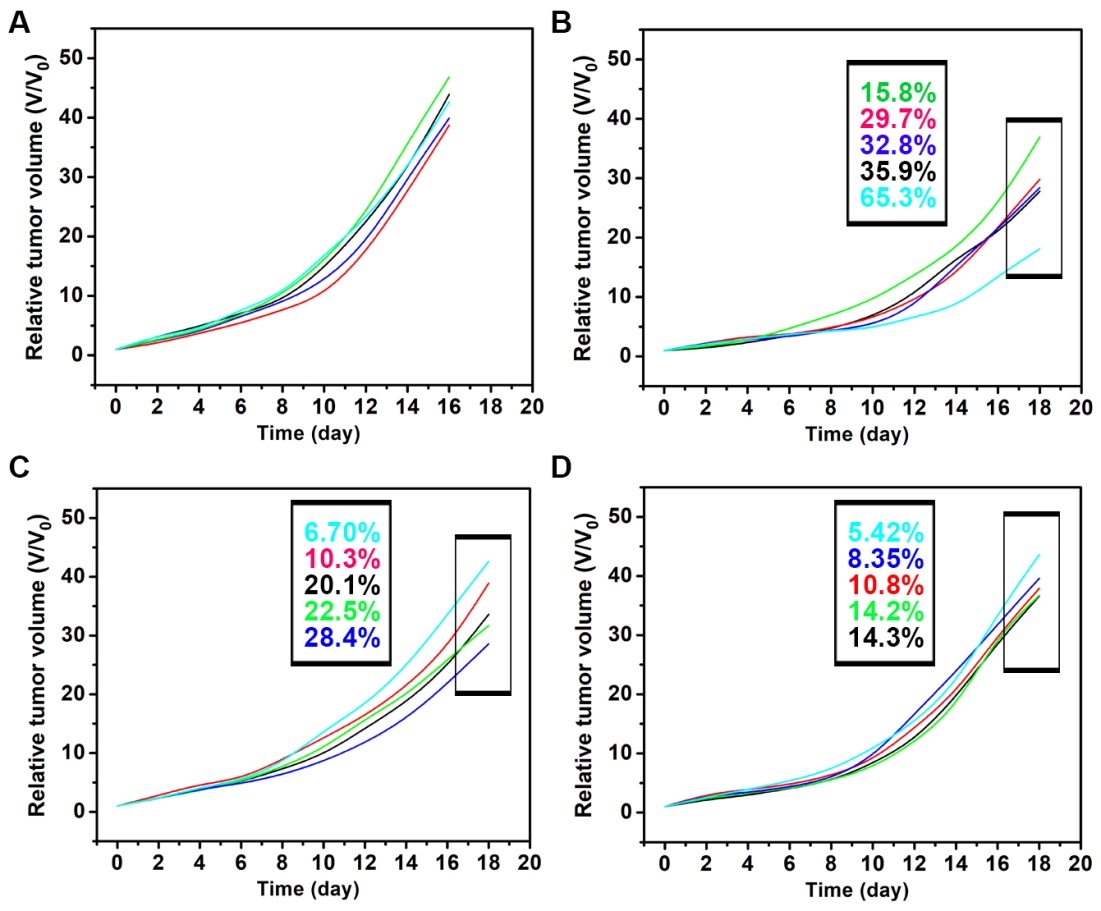


**Supplementary Fig. S36 | Individual mouse 4T1 tumor growth curves.** Mouse groups were treated with (A) control, (B) RT only, (C) RT + aLy6G, or (D) RT + DPI, respectively (n = 5 for each group). The tumor growth inhibition ratios are shown in the figure calculated by the following equation: {1 - [(exp_day18_ - exp_day0_) / (control_day16_ - control_day0_)]} * 100. This data is supplementary to the Fig. 6a in the main text.


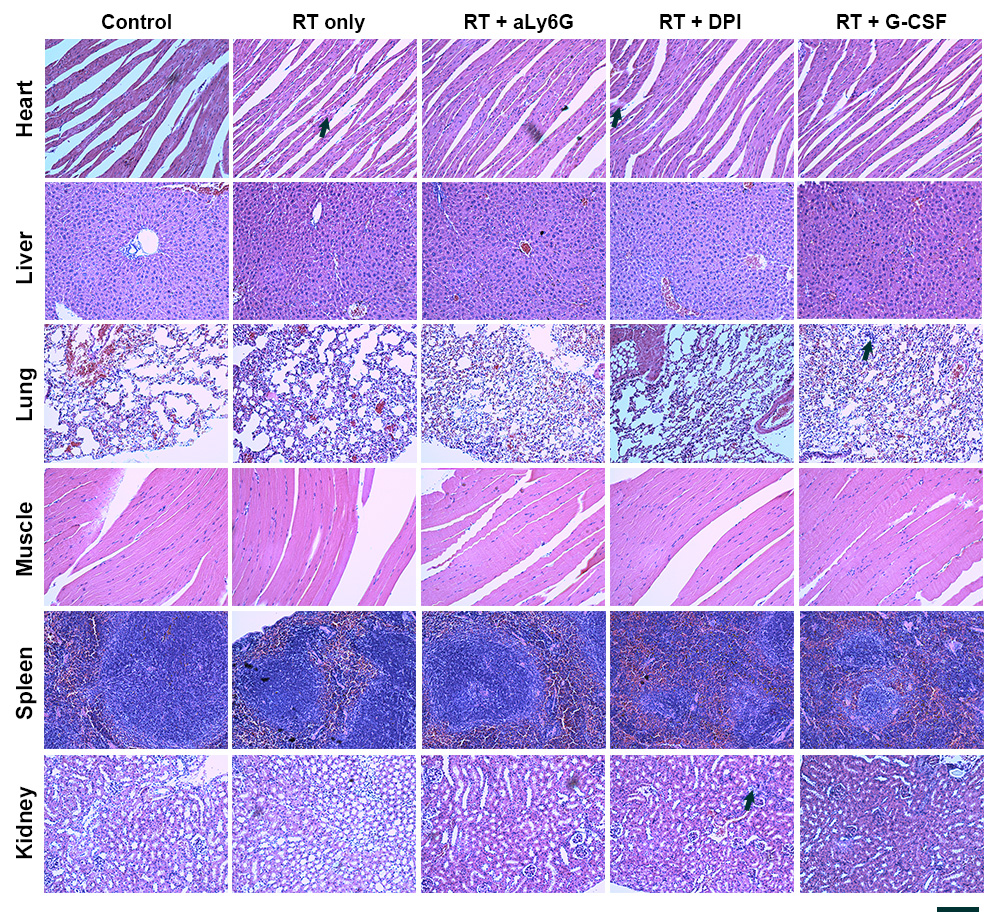


**Supplementary Fig. S37 | H&E staining of major organs after RT treatments.** Mouse organs were dissected at the end point after different treatments including control, RT only, RT + aLy6G, RT + DPI, and RT + G-CSF. Scale bar: 100 µM for all images. Black arrows indicate potential tissue damage.


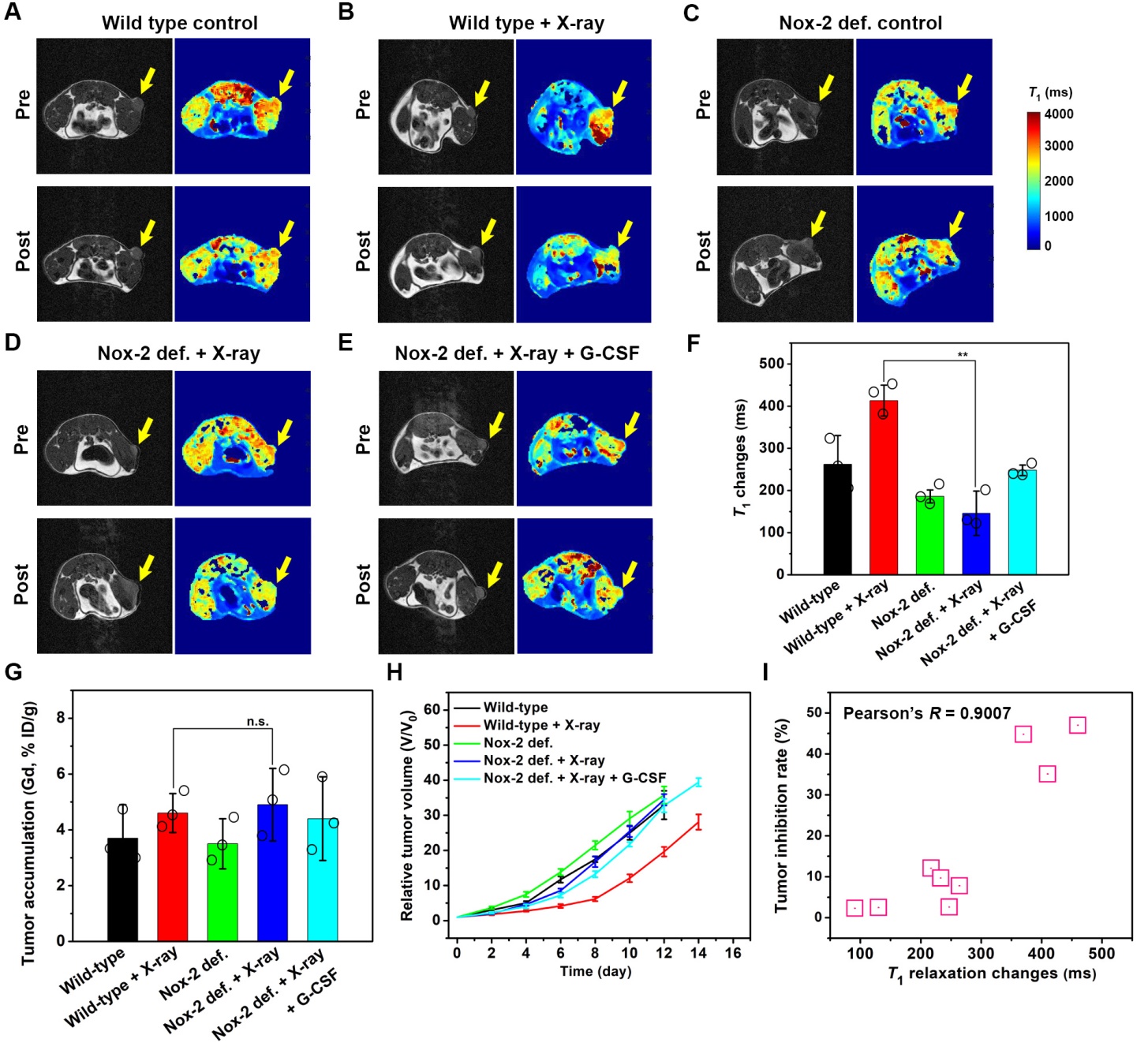


**Supplementary Fig. S38 | The aiMRI study in Nox-2 deficient mouse models with B16F10 tumors.** (A-E) The pre- and post-contrast *T*_1_ phantom images and the quantitative *T*_1_ relaxation maps of mouse tumors after receiving different treatments. The mouse groups contain wild-type control, wild-type + X-ray (15 Gy), Nox-2 deficient control, Nox-2 deficient + X-ray (15 Gy), Nox-2 deficient + X-ray + G-CSF. Yellow arrows show the mouse tumors. F, Quantitative *T*_1_ relaxation time changes in mouse tumors (n = 3, data represents mean ± s.d. **P = 0.0046, two-tailed paired t-test). G, Tumor uptake at 24 h post-injection of the IO-Gd NPs measured on the basis of Gd by ICP-AES. The n.s. indicates no significance (n = 3/group, data represents mean ± s.d.). H, The tumor growth curves of mouse groups (n = 5/group, data represents mean ± s.d.) with different treatments. The overall tumor inhibition rates are presented after each curve. I, The correlations between the *T*_1_ relaxation time change and the corresponding tumor inhibition rate for individual mouse from different treatment groups. The Pearson’s correlation coefficient *R* is 0.9007.

**Supplementary Table S1 | Summary of the *r*_1_ and *r*_2_ values of samples.** Samples include single IO NPs, IO-Gd NVs (Fe:Gd ratios of 21:1, 35.5:1, and 144:1), IO NVs, and Gd NVs. The *r*_1_ and *r*_2_ values were calculated on the basis of Gd and Fe elements for the IO-Gd samples, respectively. These values were measured at a 7 T MRI scanner based on independent triplicate experiments. Data represents mean ± s.d.

| Samples | Fe:Gd ratio | *r*_1_ (mM^-1^s^-1^) | *r*_2_ (mM^-1^s^-1^) |
| --- | --- | --- | --- |
| IO-Gd NVs | 144:1 | 1. 13 ± 0.36 | 174.5 ± 21.3 |
| IO-Gd NVs | 35.5:1 | 1.47 ± 0.31 | 195.3 ± 11.5 |
| IO-Gd NVs | 21:1 | 6.39 ± 0.49 | 113.7 ± 12.7 |
| Single IO NPs | - | 2.1 ± 0.5 | 63.5 ± 4.6 |
| IO NVs | - | 1.2 ± 0.4 | 188.4 ± 17.6 |
| Gd NVs | - | 16.3 ± 3.2 | 31.2 ± 1.5 |

**Supplementary Table S2 | Summary of the *r*_1_ and *r*_2_ values of samples after oxidation.** The IO-Gd NVs (Fe:Gd ratio of 35.5:1) were studied at different concentrations of H_2_O_2_ (1000, 500, 100 µM) and with the same amounts of NaCl and MPO. The [ox] represents for oxidation of the samples incubated with H_2_O_2_ (1000 µM), NaCl and MPO (5 U/mL). The *r*_1_ and *r*_2_ values were calculated on the basis of Gd and Fe elements for the IO-Gd samples, respectively. These values were measured at a 7 T MRI scanner based on independent triplicate experiments. Data represents mean ± s.d.

| Samples | Fe:Gd ratio | | *r*_1_ (mM^-1^s^-1^) | *r*_2_ (mM^-1^s^-1^) | |
| --- | --- | --- | --- | --- | --- |
| IO-Gd NVs, H_2_O_2_ (1000 µM) | 35.5:1 | 11.76 ± 2.1 | | | 121.7 ± 21.3 |
| IO-Gd NVs, H_2_O_2_ (500 µM) | 35.5:1 | 9.46 ± 1.5 | | | 133.1 ± 17.4 |
| IO-Gd NVs, H_2_O_2_ (100 µM) | 35.5:1 | 3.13 ± 0.44 | | | 167.6 ± 21.6 |
| IO NVs [ox] | - | 1.4 ± 0.2 | | | 117.5 ± 21.6 |
| Gd NV s [ox] | - | 10.2 ± 2.7 | | | 19.5 ± 4.3 |
